# Integrated Gut Microbiota–Drug Interaction Analysis and Network Pharmacology for the Investigation of Renal-Protective Effect of *Polygala tenuifolia* Willd

**DOI:** 10.3390/ijms262210889

**Published:** 2025-11-10

**Authors:** Jia-Chun Hu, Jian-Ye Song, Ru Feng, Meng-Liang Ye, Hui Xu, Jin-Yue Lu, Heng-Tong Zuo, Yi Zhao, Jing-Yue Wang, Jing-Yu Jin, Ling-Yu Wei, Yong-Mei Tu, Yan Wang

**Affiliations:** State Key Laboratory of Bioactive Substance and Function of Natural Medicines, Institute of Materia Medica, Chinese Academy of Medical Sciences/Peking Union Medical College, Beijing 100050, China; hujiachun@imm.ac.cn (J.-C.H.); songjianye@imm.ac.cn (J.-Y.S.); fengru@imm.ac.cn (R.F.); yemengliang@imm.ac.cn (M.-L.Y.); xuhui@imm.ac.cn (H.X.); lujinyue@imm.ac.cn (J.-Y.L.); zuohengtong@imm.ac.cn (H.-T.Z.); zhaoyi@imm.ac.cn (Y.Z.); wangjingyue12@126.com (J.-Y.W.); jennyjin7@163.com (J.-Y.J.); 13653440838@163.com (L.-Y.W.); 18783106853@163.com (Y.-M.T.)

**Keywords:** *Polygala tenuifolia* willd, gut microbiota, renal protection, metabolomics, network pharmacology

## Abstract

*Polygala tenuifolia* Willd., a widely used traditional Chinese medicine, has the function of coordinating heart and kidney and eliminating swelling. However, its renal-protective efficacy and possible material basis remain unknown. The aim of the study was to investigate the renal-protective effect of *Polygala tenuifolia* Willd. and identify the potential active substance and molecular mechanism. A gentamicin-induced kidney injury model was established to investigate efficacy. Secondly, potential active substances and molecular mechanisms were studied through integrated gut microbiota–drug interaction analysis and network pharmacology at a cellular level. Finally, 16S rRNA sequencing and untargeted metabolomics were used to elucidate the gut microbiota composition and metabolic profile change. *Polygala tenuifolia Willd.* extracts (PWE), with tenuifoliside A (TFSA) as the key compound, significantly reversed gentamicin-induced acute kidney injury in mice. The gut microbiota-derived carboxylesterase metabolized TFSA into four characteristic metabolites (M1–M4). Notably, both TFSA and M4 were detected in kidney and exerted protective effects via inhibiting TLR4–NF-κB pathway. Furthermore, metabolic pathways and gut microbiota composition change were identified. PWE treatment significantly increased the abundance of beneficial bacteria such as *Akkermansia* and *Blautia*, while reducing the abundance of harmful bacteria such as *Oscillospira*. Subsequently, PWE can reverse amino acid metabolic abnormalities by regulating the biosynthesis of phenylalanine, tyrosine, and tryptophan and ameliorating tryptophan metabolism disorder. This study was the first to verify the renal-protective effect of PWE and identify the effective substance basis (TFSA) and the molecular mechanism, providing a scientific foundation for the development of kidney drug treatment strategies targeting the intestinal flora.

## 1. Introduction

*Polygala tenuifolia* Willd. is a traditional Chinese medicine (TCM) capable of coordinating heart and kidney, calming the mind, expelling phlegm, and eliminating swelling [1,2]. Clinically, it is commonly used to treat palpitations, insomnia, cough, and phlegm [3]. Its main compounds are oligosaccharides, saponins, and organic acids [4]. Pharmacological studies have proved that tenuifoliside A (TFSA), as the main active compound in Polygala tenuifolia Willd., has many pharmacological activities such as neuroprotective, anti-depressant, and anti-inflammatory. TFSA mainly regulates the proliferation of nerve cells through ERK and PI3K, exerting anti-depression and anti-glioma activities [5,6]; TFSA can also inhibit neuro-inflammation through JNK and MAPK pathways [7]. Although there are many studies on the anti-depressant effect and mechanism of Polygala tenuifolia Willd. extracts (PWE) and its main components [8], studies of its protective effect on kidney and its possible mechanism are scarce. Moreover, studies have found that the oral bioavailability of TFSA is only 1.2% [9], which also poses new challenges to the explanation of its good efficacy.

The human gastrointestinal tract is home to tens of thousands of powerful microorganisms, known as the gut microbiota. These intestinal bacteria maintain the homeostasis of the internal environment by participating in material exchange, information transfer, and energy metabolism [10]. Due to its prominent physiological functions, intestinal flora can also affect the occurrence and development of diseases to a certain extent. More and more studies have shown that intestinal flora is an important player in kidney disease [11]. Acute kidney injury (AKI) and inflammation are also important risk factors for chronic kidney disease, which places a serious burden on society [12]. However, there is no effective treatment for AKI in the clinic, and the main strategy is symptomatic treatment with TCM. TCM is mostly administered orally, meaning it inevitably comes into contact with intestinal flora [13,14]. Drugs can stimulate intestinal flora to produce biologically active endogenous metabolites such as short-chain fatty acids (SCFAs) [15]. Meanwhile, intestinal flora can also metabolize drugs, affecting their bioavailability [16]. However, the interaction between the active component of PWE and intestinal flora is rarely reported.

Network pharmacology is a systematic approach to deciphering complex biological pathways and is an important tool for elucidating the efficacy of TCM [17,18]. Network pharmacology combines decentralized valuable information with data science to become a new approach to drug discovery [19]. In fact, NP interprets the complex interactions among compounds, targets, and diseases from the holistic perspective of multiple components, which is highly consistent with the characteristics of TCM, and can provide predictive information for the subsequent study of the mechanism.

This study verified, for the first time, the protective effect of PWE on gentamicin-induced AKI in mice. The key active ingredient TFSA was identified by an in vitro metabolic system based on high-resolution mass spectrometry and network pharmacology. Intestinal bacteria were the main metabolism site of TFSA, and the process of biotransformation by microbial carboxylesterase, including the release of four characteristic metabolites (M1–M4), was elucidated by molecular docking, inhibitor experiment, and pseudo-germ-free (PGF) mice. TFSA and M4 may exert synergistic anti-inflammatory activity by inhibiting the TLR4–NF-κB pathway and restoring gut microbiota composition and metabolic profiles. This study provides a research basis for the subsequent development of PWE, and a research paradigm and reference basis for the treatment of AKI based on intestinal bacteria.

## 2. Results

### 2.1. Renal-Protective Activity of PWE on Gentamycin-Induced AKI Mice

**Figure 1:**
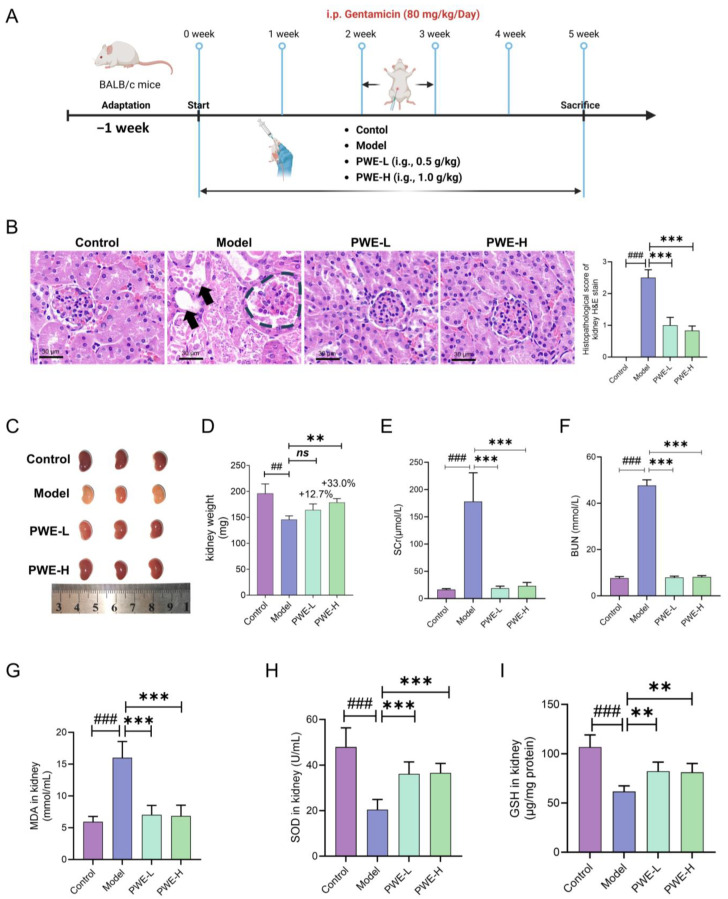
Effects of PWE on gentamycin-induced AKI. (**A**) Experimental design of gentamycin-induced AKI. (**B**) Representative images of renal H&E staining and histological score (*n* = 3). (**C**) Kidney morphological analysis (*n* = 3). (**D**) Kidney weight (*n* = 7). (**E**) Scr level (*n* = 7). (**F**) BUN level (*n* = 7). (**G**–**I**) Renal oxidative stress indicators, including MDA (**G**), SOD (**H**), and GSH. (**I**) Levels (*n* = 7). Data were presented as mean ± SD, and statistical analysis was performed with one-way ANOVA followed by Dunnett’s multiple comparisons test. Comparisons to the control group show ^##^ *p* < 0.01, ^###^ *p* < 0.001; comparisons to the model group indicate ** *p* < 0.01, *** *p* < 0.001; ns, not significant, *p* ≥ 0.05.

To investigate whether the *Polygala tenuifolia* Willd. extracts (PWE) have a renal-protective effect, an animal experiment was conducted as illustrated in Figure 1A. In this experiment, mice were administered PWE two weeks in advance, and a mouse model of AKI was constructed by intraperitoneal injection of gentamicin in the third week, with a modeling time of one week. The experiment concluded in the fifth week of administration. Following the application of H&E staining to the kidneys of mice (Figure 1B), it was observed that the glomerular structure of the model mice exhibited notable disruption, blurring of the borders, and the presence of vacuolated lesions in the renal tissues. In contrast, the tissue damage to the kidneys was found to be significantly reversed in the low and high dose groups of the PWE. The morphology and weight of the kidneys also demonstrated that the kidneys in the model group exhibited notable whitening and atrophy, whereas the kidneys underwent a notable restoration of original structure and appeared healthy once again following PWE treatment (Figure 1C,D). Serum renal function indices were examined in mice and it was found that both creatinine and urea nitrogen levels decreased significantly (Figure 1E,F, *** *p* < 0.001) after PWE treatment, but not in a dose-dependent manner. Oxidative stress-related indicators MDA, SOD, and GSH also showed significant improvement after PWE treatment (Figure 1G–I). These results demonstrate that prophylactic administration of PWE to mice can significantly alleviate the symptoms of AKI induced by gentamicin. This represents the first observation of the nephroprotective effect of PWE at the animal level.

### 2.2. Identification of Potential Renal-Protective Component of PWE

The drug–disease interaction network between PWE and AKI was analyzed by network pharmacology to investigate the potential material basis for the nephroprotective activity of PWE. A total of 58 compounds in PWE were obtained from Herb (http://herb.ac.cn/, accessed on 16 February 2025) and verified by Swiss Target Prediction (http://www.swisstargetprediction.ch/, accessed on 16 February 2025). After excluding the duplicates, we obtained 755 targets of PWE, and 9933 targets related to AKI. The Venn diagram shows 666 common targets of diseases and drugs (Figure S1A). Based on the “Centiscape 2.2” in Cytoscape 3.7.2 software, the degree, betweenness, and closeness were screened, and 134 targets greater than the median were selected. Figure S1B shows the “drug-component–core target” interrelationship. To further analyze the targets that play a significant role in AKI, the 134 core targets were submitted to the STRING database for the construction of a PPI network. The PPI network consisted of 134 nodes and 3624 edges (Figure S1C). The top ten targets for DC included TNF and NFκB1, which were consistent with inflammation.

**Figure 2:**
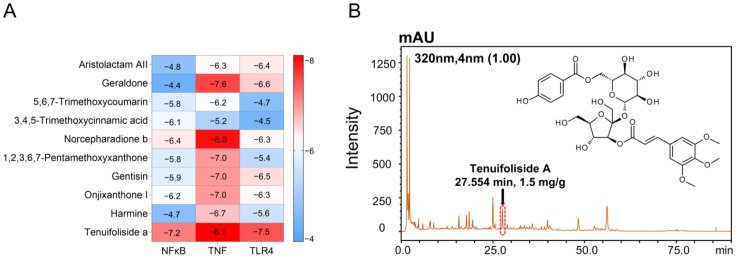
Identification of the main active substance in *Polygala tenuifolia* Willd. (**A**) Heatmap of molecular docking of potential compounds and proteins. (**B**) The HPLC fingerprint of PWE.

AKI is often accompanied by a serious inflammatory response [20]. Therefore, we selected inflammation-related TNF, NFκB1, and TLR4 as potential targets. The top 10 chemical components were selected by Cytoscape NCA, and the affinity was calculated by docking with the inflammatory target. Among the several docked compounds, TFSA exhibited the best effect (Figure 2A). And the content of TFSA in the PWE was relatively high, reaching 1.5 mg/g (Figure 2B). The chemical quality control experiments have confirmed that the content of PWE in TFSA remains stable across different batches (Figure S2). Therefore, TFSA may be key to nephroprotective efficacy as an active component of PWE.

### 2.3. Microbial Carboxylesterase Mediated the Biotransformation of TFSA

**Figure 3:**
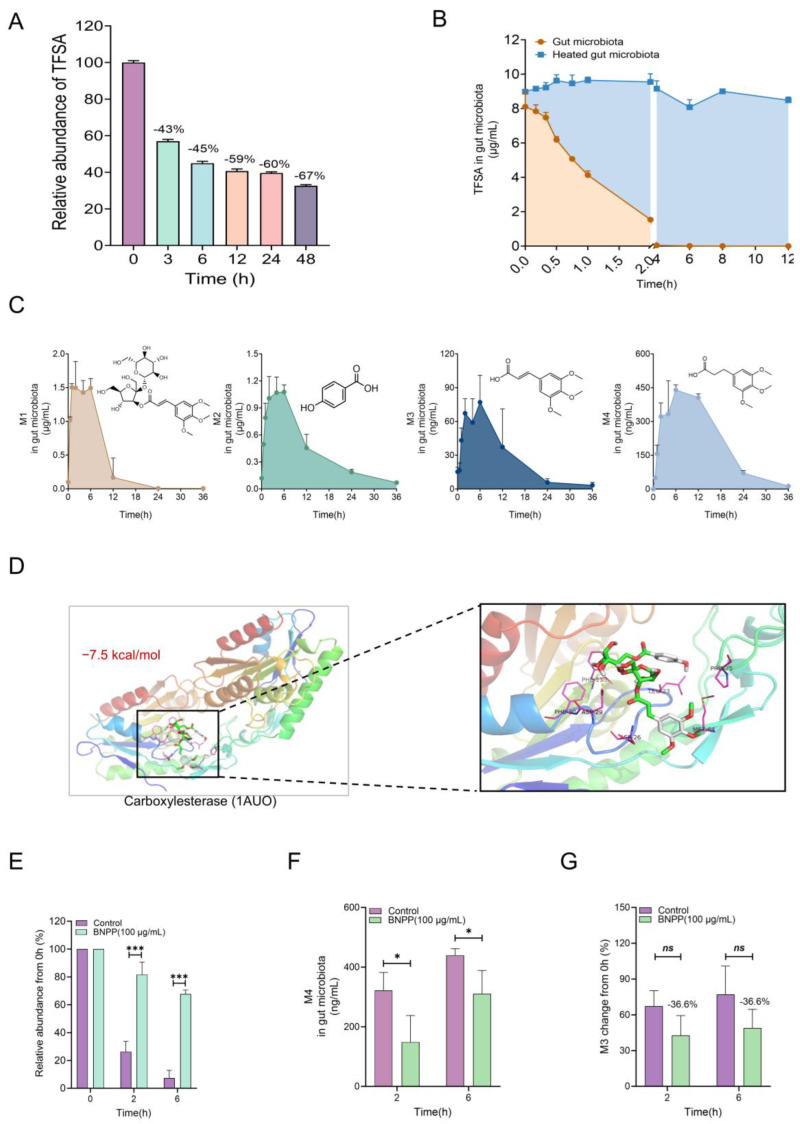
Carboxylesterase in the gut microbiota mediates the biotransformation of TFSA. (**A**) Relative abundance of TFSA in PWE in the in vitro gut microbiota metabolic system. (**B**) In vitro metabolic curve of TFSA in the gut microbiota. (**C**) Quantitative analysis of M1–M4 in gut microbiota. (**D**) Molecule docking between TFSA and carboxylesterase (1AUO). (**E**–**G**) The abundance of TFSA (**E**), M3 (**F**), and M4 (**G**) by incubation with bis-*p*-nitrophenyl phosphate (BNPP) in vitro (*n* = 3). Data were presented as mean ± SD, and a two-tailed Student’s *t* test was used for analysis (* *p* < 0.05, *** *p* < 0.001; ns, not significant, *p* ≥ 0.05).

Oral medications inevitably interact with gut microbiota, thereby resulting in low bioavailability of many natural substances. We tested the stability of TFSA from PWE in three incubation systems, including simulated gastric fluid (Figure S3A), artificial intestinal juice (Figure S3B), and gut microbiota (Figure 2A). The results indicated that TFSA could be significantly metabolized by the intestinal flora. In order to further verify whether TFSA can interact with gut microbiota, stability experiments were carried out using the standard compound (Figure 3B and Figure S3C–E). As shown in Figure S2E, TFSA was clearly not metabolized in the in vitro liver homogenate system. In contrast to that, TFSA was completely degraded by intestinal flora within 4 h, while the negative control group was not metabolized, suggesting that intestinal flora played an important role in the biotransformation of TFSA (Figure 3B). It can be concluded that intestinal bacteria are the main site of TFSA metabolism, rather than the liver.

Further experiments were conducted by LC/MS-Q-TOF to identify the metabolites of TFSA generated by the gut microbiota. A rapid LC-MS/MS analysis method was established to accurately quantify TFSA and metabolites in the in vitro metabolic system. The LC-MS/MS information of all the above metabolites is shown in Table 1 and Figure S3F. Figures S4–S6 and Table S1 show the extracted ion chromatograms, mass spectra, and speculated fragmentation patterns of M1, M3, and M4. Due to its small molecular weight and low abundance, M2 was not able to extract the corresponding ion chromatogram in high-resolution mass spectrometry, but the metabolic curve could be detected in the quantification of low-resolution mass spectrometry after purchasing standard products.

Ester bonds exist in the chemical structure of TFSA, and the metabolites M2, M3, and M4 are also the products of ester bond hydrolysis. Therefore, we investigated the effect of gut microbiota-derived carboxylesterase on TFSA biotransformation. Firstly, the binding process of TFSA and the carboxylesterase active pocket was simulated using virtual molecular docking. Figure 3D shows the 3D docking schematic diagrams of TFSA and carboxylesterase. The binding affinity between the two molecules was relatively stable (−7.5 kcal/mol). In addition, after the carboxylesterase inhibitor BNPP (100 μg/mL) was added to the in vitro gut microbiota metabolic system, the metabolism of TFSA (Figure 3E) and the generation of M4 (Figure 3F) were inhibited significantly, while M3 production did not yield statistically significant differences, it did demonstrate a discernible tendency towards inhibition, with a reduction of 36.6% observed at both 2 h and 6 h points (Figure 3G). Taken together, the evidence from this study demonstrates that microbial carboxylesterase serves as a critical mediator in TFSA biotransformation.

Having established the in vitro metabolic profile of TFSA, the critical question of its in vivo interaction with gut microbiota was subsequently addressed using normal and PGF mice. The colony count results showed that the inhibition rates of aerobic bacteria and anaerobic bacteria reached 85.6% and 90.1%, respectively, indicating that the PGF state had been achieved (Figure S7). Fecal excretion experiment showed that the content of TFSA at 12 h (*** *p* < 0.001) in the stool of PGF mice was significantly higher than that in the normal group, and the metabolites showed an opposite trend. The content of M1 in the stool of the PGF group was not significant compared with the normal group at 12 h, while M2 (*** *p* < 0.001), M3 (*** *p* < 0.001), and M4 (*** *p* < 0.001) were lower than that in the normal group at 12 h (Figure S8). These results indicate that TFSA may alleviate kidney injury by synergizing with its gut microbiota-derived metabolites.

### 2.4. TFSA and Its Metabolites Ameliorate AKI Based on NF-κB Pathway via Network Pharmacology Analysis

**Figure 4:**
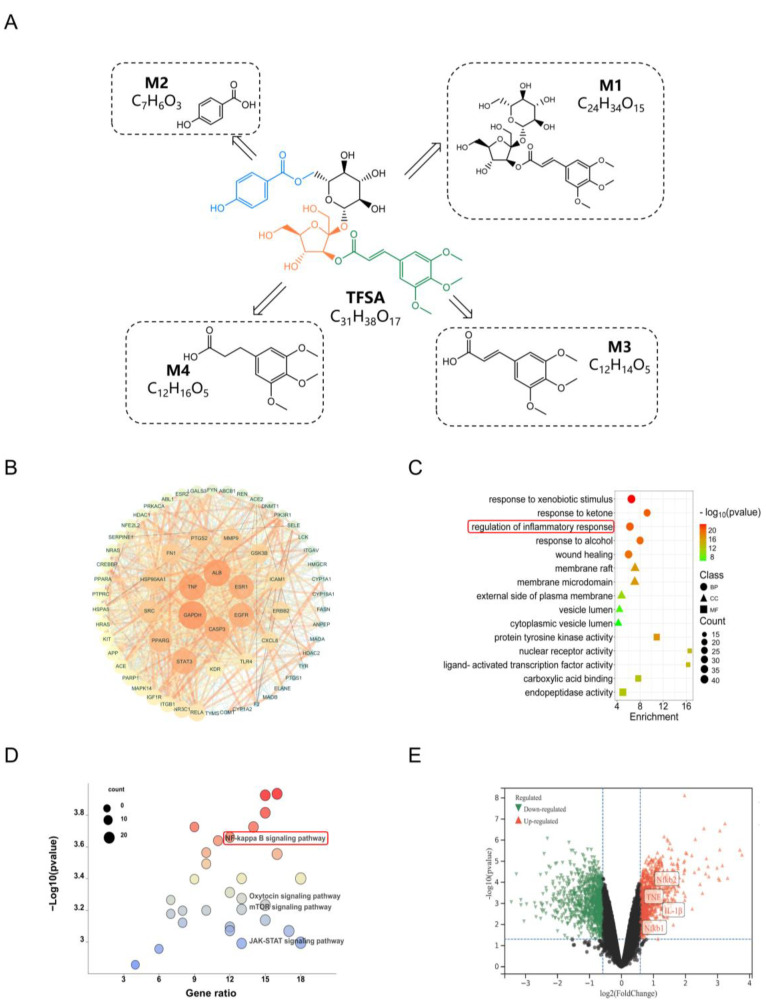
TFSA and its metabolites ameliorate AKI based on the inflammatory pathway by network pharmacology. (**A**) The chemical structure of TFSA, M1, M2, M3, and M4. (**B**) PPI network of the core target. (**C**) GO enrichment analysis. The top 5 terms of BP, CC, and MF were displayed in a hierarchical network diagram (The darker the red color, the stronger the correlation; the darker the blue color, the weaker the correlation). (**D**) KEGG pathways analysis. Top 30 pathways were displayed in a bubble plot. (**E**) Validation of the differentially expressed genes of AKI using GEO datasets (Green: Down-regulated, Red: Up-regulated).

TFSA can be mediated by intestinal bacteria to produce four metabolites (Figure 4A). To further investigate the relationship between TFSA, metabolites, and AKI, network pharmacology was used to reveal underlying mechanism. The targets of TFSA and M1–M4 were retrieved by Swiss Target Prediction, and 327 relevant targets were obtained after removing duplicates. Consistent with previous findings, 9933 targets were related to AKI. A total of 288 common targets of drug and AKI were identified, as shown in (Figure S9A).

A prototype drug and its metabolites–targets network was shown in Figure S9B, the network consisted of 277 nodes and 437 edges. Based on Centiscape 2.2, 65 targets were selected as core targets. The PPI network of core targets was visualized using Cytoscape 3.7.2 (Figure 4B). The core targets included TNF, EGFR, and TLR4. These targets are closely related to the inflammatory response, consistent with our previous findings.

In the results of GO enrichment analysis, the top 5 pathways sorted by their *p* values were visualized (Figure 4C). The BP terms focused on response to xenobiotic stimulus, response to ketone, regulation of inflammatory response, response to alcohol and wound healing. Among the enriched Cellular Component terms were membrane rafts, microdomains, the external plasma membrane surface, and vesicular lumina. Molecular Function analysis showed over-representation of protein tyrosine kinase activity, nuclear receptor function, ligand-activated transcription factor activity, carboxylic acid binding, and endopeptidase activity. Bubble chart visualization of the top 30 KEGG pathways (by *p*-value) demonstrated that the NF-κB, Oxytocin, mTOR, and JAK-STAT signaling pathways emerged as critically involved in therapeutic response to AKI (Figure 4D). Notably, the NF-κB signaling pathway were visualized by “pathview”, highlighting the crucial role of the inflammatory response in AKI (Figure S10). Meanwhile, four targets from GSE273063 dataset that are related to the inflammatory response (TNF, IL-1β, NfκB1 and NfκB2) all up-regulated in AKI. (Figure 4E). The result was consistent with our previous analysis, suggesting that TFSA and its metabolites may ameliorate AKI by modulating the NF-κB pathway.

### 2.5. TFSA and M4 Alleviate the Inflammatory Response by Targeting TLR4 and NF-κB

**Figure 5:**
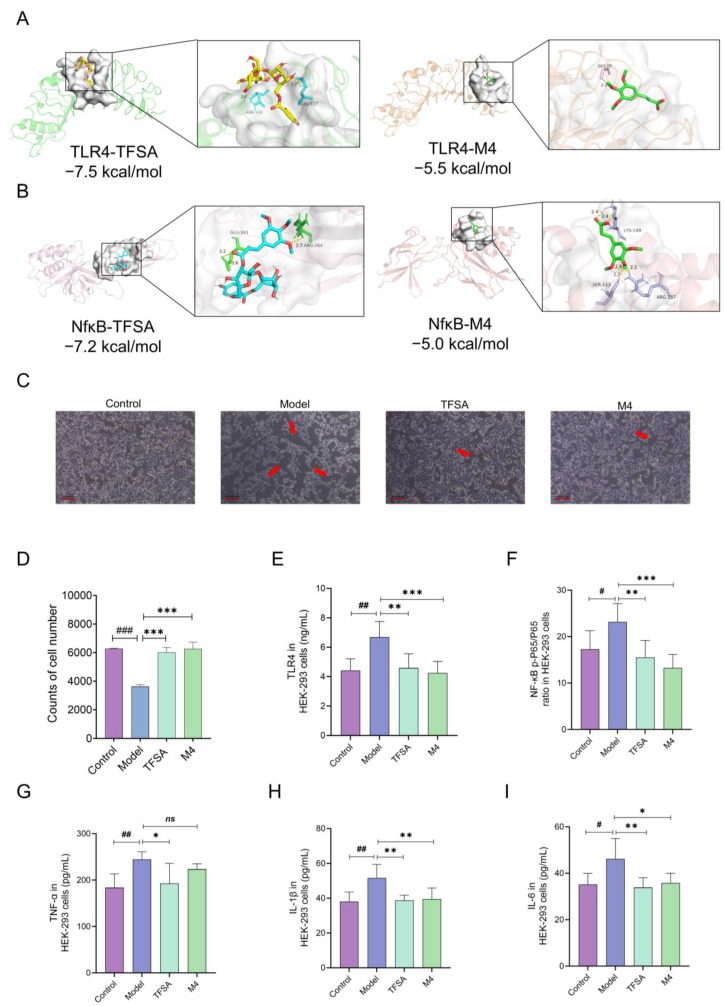
TFSA and M4 inhibit the TLR4–NF-κB pathway to exert renal-protection activity at the cellular level. (**A**) Molecular docking of TFSA/M4 with TLR4 (2Z62). (**B**) Molecular docking of TFSA/M4 with NF-κB (1SVC). (**C**) Representative images of cell density after treatment. (**D**) The counts of cell numbers. (**E**) TLR4 levels in HEK293T cells after treatment. (**F**) NF-κB p-P65/P65 levels in HEK293T cells after treatment. (**G**–**I**) Inflammatory indicator levels in HEK293T cells after treatment, including TNF-α (**G**), IL-1β (**H**), and IL-6 (**I**) (*n* = 3). Data are presented as mean ± SD, and statistical analysis was performed with one-way ANOVA followed by Dunnett’s multiple comparisons test. Comparisons to the control group show ^#^ *p* < 0.05, ^##^ *p* < 0.01, ^###^ *p* < 0.001; comparisons to the model group indicate * *p* < 0.05, ** *p* < 0.01, *** *p* < 0.001; ns, not significant, *p* ≥ 0.05.

In the normal and PGF mice experiment, TFSA and M4 were detected in kidney samples at 12 h, indicating that they may target the kidney to exert nephroprotective effects (Figure S6B). Furthermore, we docked TFSA and M4 with NfκB and TLR4. The docking results showed that the compounds and targets had good binding ability (Figure 5A,B).

To prove whether TFSA and M4 can improve AKI by targeting NfκB and TLR4, HEK-293 cells were subjected to inflammation modeling using LPS, TFSA and M4 were given, respectively. As can be seen from the microscope images and cell counts (Figure 5C,D), the number of cells in the Model group was significantly reduced, and this phenomenon was significantly reversed after administration of TFSA and M4 (*** *p* < 0.001). As shown in Figure 5E,F, it was found that both TFSA and M4 significantly inhibited the activation of TLR4 (TFSA: ** *p* < 0.01; M4: *** *p* < 0.001) and NF-κB (TFSA: ** *p* < 0.01; M4: *** *p* < 0.001) in inflammatory states. For the activation of NF-κB, we also conducted verification using Western blotting in HK-2 cells. Similarly, it was found that TFSA and M4 could significantly inhibit the phosphorylation of NF-κB (Figure S11). The levels of downstream inflammatory factors like TNF-α (TFSA: * *p* < 0.05), IL-1β (TFSA: ** *p* < 0.01; M4: ** *p* < 0.01), and IL-6 (TFSA: ** *p* < 0.01; M4: * *p* < 0.05) were also significantly down-regulated (Figure 5G–I). These results point to the TLR4–NF-κB signaling axis as a potential target for TFSA and M4 in mediating their anti-inflammatory activity.

### 2.6. Effects of PWE on the Gut Microbiota of AKI Mice

**Figure 6:**
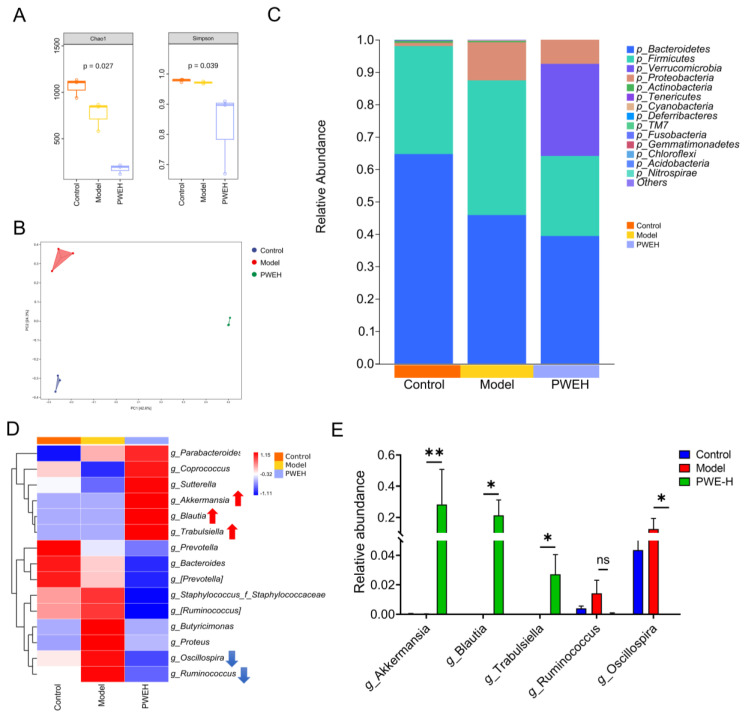
Effects of PWE on gut microbiota in AKI mice. (**A**) α-diversity of gut microbiota in mice. (**B**) β-diversity of gut microbiota in mice. (**C**) Relative abundance of gut microbiota at the phylum level. (**D**) Heatmap of the gut microbiota in mice at the genus level. (**E**) Relative abundance of specific species at the genus level. Data are presented as mean ± SD, *n* = 3, and statistical analysis was performed with one-way ANOVA followed by Dunnett’s multiple comparisons test (* *p* < 0.05, ** *p* < 0.01; ns, not significant, *p* ≥ 0.05).

The above section explored the effect of gut microbiota on the efficacy of PWE, and we wondered whether PWE could affect gut microbiota as well, so we collected feces from three groups (Control, Model, and PWE-H) of mice for 16S rRNA sequencing analysis. As shown in Figure 6A,B, the α diversity of the PWE-H group was significantly reduced, and the β diversity also showed that the gut microbiota composition of the PWE-treated mice was significantly different from the other two groups, which indicated that PWE could significantly regulate the structure of gut microbiota. Further analysis of the gut microbiota’s taxonomic composition across groups was conducted. The 15 most abundant bacterial phyla were selected and visualized in ascending order based on their relative abundance (Figure 6C). At the phylum level, Firmicutes and Bacteroidetes accounted for the highest proportion. After PWE treatment, Firmicutes decreased, while Verrucomicrobia significantly increased.

As the genus level more accurately reflects treatment-specific microbiota shifts, the top 15 genera were selected and plotted in descending order of relative abundance (Figure 6D). Further analysis revealed that PWE administration markedly elevated beneficial bacteria (e.g., *Akkermansia*, *Blautia*), while counteracting the model-induced proliferation of *Oscillospira* (Figure 6E). The correction of this microbial imbalance suggests that microbiota restoration constitutes a potential mechanism for the renoprotective effects of PWE.

### 2.7. Effects of PWE on Metabolic Disorders in AKI Mice

**Figure 7:**
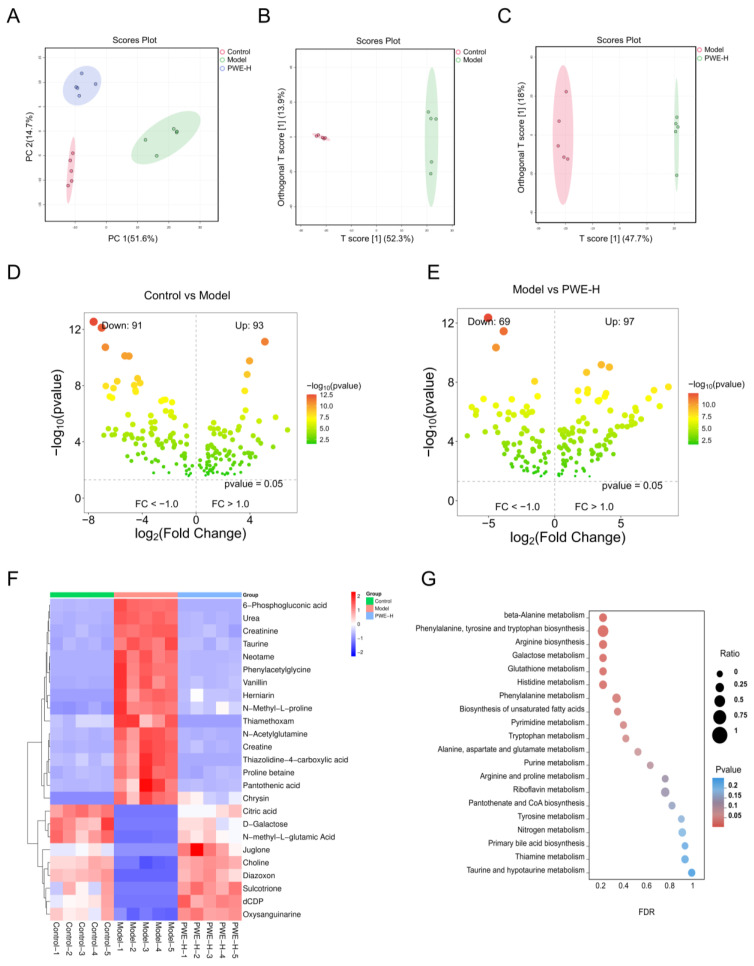
Effects of PWE on metabolic profile in AKI mice. (**A**) PCA showing the distribution trends of all groups (*n* = 5). (**B**) OPLS-DA analysis of the Control group and the Model group (*n* = 5). (**C**) OPLS-DA analysis of the Model group and the PWE-H group (*n* = 5). (**D**) Volcano plots of differential metabolites for the Control group and the Model group. (**E**) Volcano plots of differential metabolites for the Model group and the PWE-H group. (**F**) Heatmap clustering of top 25 differential metabolites (*n* = 5). (**G**) KEGG enrichment analysis of 146 differential metabolites.

A dynamic interplay exists between gut microbial community structure and host metabolic homeostasis. Thus, an untargeted metabolomics approach was applied to serum samples obtained from mice to examine the metabolic impacts of PWE on AKI. PCA and OPLS-DA were performed to explore the separation among different groups (Figure 7A–C). PCA is a dimensionality reduction technique that reduces the dimensionality of the dataset while preserving the most important statistical information. The results revealed clear intergroup separation and substantial intragroup aggregation in the control, model, and PWE-H groups. Comparing the control group and model groups, 93 metabolites were up-regulated and 91 were down-regulated (Figure 7D). Similarly, 97 were up-regulated and 69 were down-regulated between the model and PWE-H groups (Figure 7E). The intersection of these two conditions resulted in 146 differential metabolites. Based on the *p*-value, the top 25 differential metabolites, including taurine, phenylacetylglycine, and N-acetylglutamine, were identified as key metabolites in this study (Figure 7F). Enrichment analysis of the 146 differential metabolites via KEGG revealed the 20 most relevant metabolic pathways, which included phenylalanine/tyrosine/tryptophan/arginine biosynthesis, beta-alanine and glutathione metabolism, among others. The significant representation of amino acid metabolism-related pathways indicates that PWE’s correction of metabolic disorders in AKI mice occurs primarily through regulating these processes (Figure 7G).

## 3. Discussion

AKI has a hidden clinical onset and diversified clinical manifestations. With the progress of the disease, if it is not effectively controlled, it is very likely to develop into chronic kidney disease or end-stage renal disease, which brings a huge economic burden to individuals and society [21]. At present, AKI lacks targeted drugs; conventional immunosuppressants and antihypertensive drugs are still the main therapeutic regimen, which have inevitable side effects. Therefore, the development of therapeutic drugs for AKI has great clinical significance and market value.

TCM is a great treasure trove for drug development [22,23,24]. Many studies have shown that active ingredients in traditional Chinese medicine have significant effects on kidney diseases [25,26,27]. *Polygala tenuifolia* Willd. has the effect of calming the mind, removing phlegm, and eliminating swelling. Clinically, it is often used to treat insomnia and anxiety, and there are Chinese patent medicines and Chinese herbal compound preparations with *Polygala tenuifolia* Willd. as the main component for the auxiliary treatment of kidney disease. Here, we report for the first time that prophylactic oral administration of PWE significantly protected kidneys from damage caused by gentamicin in mice.

Multiple studies have reported that inflammation-related pathways may be potential targets for the treatment of AKI. Based on network pharmacology prediction of the active components in PWE, we hypothesized that the nephroprotective effects of PWE are related to inflammatory targets such as TNF, TLR4, and NF-κB. Molecular docking of the top 10 ranked compounds in PWE with these targets revealed that TFSA exhibited the strongest binding affinities to all three inflammatory targets. Due to the possible variations in the content of TFSA in different batches of PWE [28], we conducted chemical quality control on the purchased PWE and it was found that the content of TFSA was stable (Figure S2), which was suitable for subsequent in vitro and in vivo experiments.

TFSA has been documented to demonstrate a broad spectrum of pharmacological properties, including anti-inflammatory and antioxidant activities, as well as efficacy against depression and tumors [6,29]. However, studies on the mechanism of renal-protective action of TFSA are rare. Furthermore, the oral bioavailability of TFSA is only 1.17%, and its interaction with intestinal bacteria remains poorly understood [9]. In our study, we found that TFSA could be significantly metabolized by the intestinal flora, which may be the reason for its low bioavailability. Metabolic experiments in vivo and in vitro proved that intestinal flora is the main site of TFSA biotransformation, rather than the liver. Then, LC/MS-Q-TOF was used to identify the possible metabolites M1–M4 in the intestinal flora of TFSA, and quantitative analysis was conducted. It was found that M1–M4 were all intermediate metabolites, and completely metabolized by gut microbiota at 36 h. The metabolic reaction of TFSA mediated by intestinal flora mainly includes hydrolysis of the carboxylate bond, hydrolysis of the glycoside bond, and reduction of the double bond. M1 and M2 are the two parts after the break of the carboxylate bond, respectively. M1 is glomeratose A, a compound isolated from the rhizome of Polygala tenuifolia Willd., which has the activity of inhibiting lactate dehydrogenase, and may be the material basis for the anti-tumor efficacy of TFSA [30]. The other part of M2 is p-hydroxybenzoic acid, often used as a food additive, which has a wide antibacterial spectrum. Moreover, pharmacological studies have shown that M2 can maintain intestinal barrier homeostasis in a microbiota-dependent manner, thus producing the effect of improving colitis [31]. M3 is the product of M1 after the removal of two monosaccharides, and the metabolite M4 is produced after the reduction of the M3 double bond. M3 is (E)-3-(3,4,5-trimethoxyphenyl) acrylic acid, M4 is 3-(3,4,5-trimethoxyphenyl) propanoic acid. M3 has been reported to possess antitumor, antiviral, and central nervous system (CNS) activities [32]. However, there are not many studies on M4 in the previous literature reports, and further studies are needed to explore their biological activities.

To better reveal the metabolic process of TFSA in the intestinal bacterial system, we speculated that the carboxylesterases derived from the intestinal flora might be the key enzymes in TFSA metabolism based on the types of metabolic reactions. Virtual molecular docking was used to predict the binding process of TFSA and the carboxylesterase 1AUO active pocket from the computer simulation level, and it was more excreted in its prototype form found that the two have good binding energy. Subsequently, BNPP, a carboxylesterase inhibitor, was added to the intestinal bacteria metabolism system in vitro, and it was found that TFSA metabolism was significantly inhibited. Therefore, we can conclude that the carboxylesterase derived from intestinal bacteria mediates the biotransformation of TFSA.

The gut microbiota-mediated biotransformation of TFSA has been confirmed in vitro, and we further validated the crucial role of the gut microbiota in vivo using a PGF mouse model. In the fecal excretion experiment, except that TFSA of the Control group in feces was higher than that in the PGF group, metabolites were reduced in the PGF group, indicating that TFSA was more excreted in prototype form without metabolism in the absence of intestinal bacteria. The kidney distribution experiment showed that TFSA and metabolite M4 could be detected in the kidney, indicating that these two substances may be the basis of its renal-protective efficacy, and their contents in the PGF group were significantly lower than those in the normal group, which further indicated that intestinal flora had a significant contribution to the biotransformation and pharmacodynamic activity of TFSA. This part of the experiment needs to be further studied at the level of single bacteria. However, the above experimental results can enlighten us that the intestinal bacteria and their functional metabolic enzymes may be expected to become the key mediators for the release of prodrugs or the key targets for the action of healthy food, and provide the basis for subsequent product development.

We used network pharmacology to screen the targets where TFSA and four metabolites overlap with AKI. Two targets (TLR4 and NF-κB), which are highly correlated with inflammation, have emerged as key mechanisms underlying the treatment of TFSA. It is worth noting that these two targets also correspond to the previous network pharmacology results of PWE. The results of the tissue distribution experiment confirmed that only TFSA and metabolite M4 could enter the kidneys and exert their effects directly. Therefore, we investigated the binding capabilities of both to the target protein. The docking results of TFSA and M4 with NF-κB and TLR4 indicated that the compounds and targets had good binding ability. Hence, we investigated the influence of the cellular level on protein expression. At the cellular level, TFSA and M4 were both found to effectively inhibit the overactivation of the TLR4–NF-κB pathway and down-regulate the levels of a series of inflammatory factors. These results demonstrate that TFSA, the active components of PWE, is transformed into M4 by the gut microbiota and synergistically exerts renal-protective effects by inhibiting the TLR4–NF-κB pathway.

These results demonstrated the reno-protective effects of active metabolites produced by the biotransformation of PWE by gut microbiota. We were also interested in whether PWE affected gut bacteria. As this is a preliminary exploratory experiment, we chose 16S rRNA sequencing due to technical and cost advantages. The results indicated that PWE treatment could significantly change the gut microbiota structure of AKI mice, especially the upregulation of *Akkermansia*. As a widely studied probiotic, *Akkermansia* can produce a variety of beneficial metabolites (such as SCFAs) and maintain the intestinal barrier, and these beneficial effects may also contribute to the therapeutic effects of PWE on AKI mice [33]. *Oscillospira*, which is significantly increased in model mice, has been reported to be a potential pathogen responsible for producing LPS [34], and PWE treatment can significantly reduce its abundance, but the specific mechanism needs to be further explored. Although from an overall structural perspective, the intervention of PWE led to a decrease in the α diversity of the mouse microbiota. However, when we look at the specific changes in bacterial genera, this seems to be a sign of the community actively reconstructing itself from a pathological state to a healthy state, with the abundance of beneficial bacteria increasing while that of harmful bacteria decreasing. And the overall metabolic function of the community has also been improved. Not only that, other natural drugs that interact significantly with the microbiome, such as berberine, also showed a decrease in diversity after intervention, but their efficacy was remarkable [12]. Therefore, we believe that the changes in the diversity of intestinal bacteria must be interpreted in conjunction with the specific biological context and functional output. Since this was an exploratory experiment, we examined the overall effect of PWE on the intestinal bacteria. In the future, we will also conduct further investigations into the candidate compounds that cause changes in the structure of the intestinal microbiota.

The changes in gut microbiota structure are closely related to the metabolic profile of the host. Thus, metabolomics analysis was employed to further elucidate the regulatory mechanisms of PWE in the metabolic profile. KEGG enrichment analysis showed that PWE could regulate amino acid metabolism pathways in AKI mice, such as beta-alanine metabolism, phenylalanine, tyrosine, and tryptophan biosynthesis. Differential metabolite analysis revealed changes in taurine, phenylacetylglycine, N-acetylglutamine, and other metabolites. Previous studies have shown these changes in metabolites could be responsible for kidney injury. In particular, phenylacetylglycine, a gut microbiota-derived metabolite associated with cardiovascular toxicity [35], was enriched in the model group and decreased after PWE treatment, suggesting it may serve as a potential biomarker for the nephroprotective effect of PWE. The interaction between PWE and the intestinal microbiota involves two aspects: on one hand, TFSA is directly metabolically activated and becomes effective; on the other hand, PWE can also promote the remodeling of the microbial community structure. The two aspects work together to exert a protective effect on the kidneys.

## 4. Materials and Methods

### 4.1. Chemicals and Reagents

PWE was purchased from Shanghai yuanye Bio-Technology Co., Ltd. (Shanghai, China). Gentamicin was purchased from Shanghai Macklin Biochemical Technology Co., Ltd. (Shanghai, China). TFSA, glomeratose A, 4-hydroxybenzoic acid, (E)-3-(3,4,5-trimethoxyphenyl) acrylic acid, 3-(3,4,5-trimethoxyphenyl) propanoic acid, glipizide, phosphate-buffered saline (PBS), bis-p-nitrophenyl phosphate (BNPP), erythromycin, oxytetracycline, cefadroxil, and anaerobic medium were purchased from Solarbio Biotechnology, Co., Ltd. (Beijing, China). Phosphoric acid, propionic acid, hexanoic acid, isovaleric acid, and butyric acid were purchased from Sigma-Aldrich (St. Louis, MO, USA). The purity of the above chemicals was greater than 98%. Chromatography-grade methanol and acetone were obtained from Thermo Fisher Scientific Inc. (Fair Lawn, NJ, USA). Other reagents were obtained from domestic reagent companies.

### 4.2. Instruments and Methods

Quantitative analysis of TFSA and its metabolites was conducted on a liquid chromatograph coupled with a triple quadrupole mass spectrometer LCMS-8050 (Shimadzu, Japan). Chromatographic separation was achieved using an Alltima C18 column (4.6 mm × 150 mm, 5 μm; GRACE^®^, Columbia, MD, USA) maintained at 40 °C, with a mobile phase consisting of deionized water (A) and methanol (B) at a flow rate of 0.4 mL/min. The autosampler temperature was set to 4 °C. The gradient elution program was set as follows: 10% B (0–0.5 min), 10–95% B (0.5–6 min), and 95–10% B (10–12 min). Mass spectrometric detection was performed in multiple reaction monitoring (MRM) mode under negative ionization, monitoring the transitions m/z 681.25→137.10 (TFSA), 561.35→237.15 (M1), 137.15→92.95 (M2), 237.20→103.15 (M3), 239.25→180.10 (M4), and 444.15→319.15 (glipizide, IS). The gas flow rates were set at 3.0 L/min (nebulizing gas), 10 L/min (drying gas), and 10 L/min (heating gas).

Structural identification of TFSA metabolites was performed using liquid chromatography coupled with quadrupole time-of-flight tandem mass spectrometry LC/MS-Q-TOF (Shimadzu, Japan). Chromatographic separation was achieved using an Alltima C18 column (4.6 mm × 150 mm, 5 μm; GRACE^®^, USA) maintained at 40 °C with a mobile phase consisting of deionized water (A) and methanol (B) at a flow rate of 0.4 mL/min. The gradient elution program was optimized as follows: 10% B (0–1 min), 10–95% B (1–20 min), and 95–10% B (20–30 min). The autosampler temperature was maintained at 4 °C, and detection was performed at 320 nm using a photodiode array detector. Mass spectrometric conditions included: heating block temperature of 200 °C, nebulizing gas flow rate of 1.5 L/min, detector voltage of 1.8 kV, and collision energy of 70%. Automated fragmentation was performed with m/z scanning ranges of 50–1000 for both primary and secondary ions.

An analytical balance (Mettler-Toledo, Switzerland), a vortex mixer (Vortex-Genie 2, USA), a high-speed centrifuge (Eppendorf, Hamburg, Germany), and an incubator shaker (Shanghai Longyue Co., Ltd., Shanghai, China) was used.

### 4.3. Animals

Eight-week-old male Sprague–Dawley rats and Balb/c mice were obtained from Beijing HFK Bioscience Co., Ltd., Riyadh, Saudi Arabia. The animals weighed 200–300 g (rats) and 20–25 g (mice), respectively. Housing conditions were maintained at 22–24 °C with 40–60% relative humidity under a 12 h light/dark cycle. All animals received a standard diet and water ad libitum. The experimental protocol received ethical approval from the Laboratory’s Institutional Animal Care and Use Committee of the Chinese Academy of Medical Sciences and Peking Union Medical College (Approval No.: 00009913).

### 4.4. Renal-Protection Efficacy of PWE

We mainly determined the sample size through literature [36,37], and also comprehensively considered the ethical principles (following the 3R principle), the availability of animal resources, and the feasibility of experimental procedures. The experimental design was as follows: Prior to efficacy assessment, all mice underwent an overnight fast with ad libitum access to water. The experimental design comprised four groups (*n* = 7 per group): (1) Control group: received daily normal saline; (2) Model group: administered gentamicin (80 mg/kg, i.p.) daily; (3) PWE-Low group: treated with PWE (0.5 g/kg, i.g.) daily; (4) PWE-High group: receiving PWE (1 g/kg, i.g.) daily. Upon completion of the study, all animals were euthanized under anesthesia induced by 0.5 mL of 20% (*v*/*v*) urethane. Body weights were recorded, and samples of feces, kidneys, and blood were collected for subsequent analysis.

### 4.5. Metabolism of TFSA in Normal and PGF Mice

For kidney distribution and fecal excretion assessment in normal mice, animals were fasted overnight with free access to water prior to the experiment. Five Balb/c mice received a single oral dose of TFSA (30 mg/kg). 12 h post-administration, the mice were anesthetized using 20% (*v*/*v*) urethane and subjected to systemic perfusion with saline. Kidney and fecal samples were then collected and homogenized in a 3-fold volume of normal saline. Aliquots of tissue homogenate (100 μL) were mixed with 300 μL of methanolic glipizide solution (100 ng/mL) as an internal standard, followed by vortexing for 30 s and centrifugation at 15,000 rpm for 15 min. The resulting supernatant (3 μL) was subsequently analyzed by LC-MS/MS.

To establish a pseudo-germfree (PGF) model, five Balb/c mice received a three-day combined antibiotic regimen consisting of erythromycin (200 mg/kg/day), oxytetracycline (200 mg/kg/day), and cefalexin (100 mg/kg/day) via oral administration [25]. All subsequent procedures were consistent with those described above.

### 4.6. Cell Culture and Modeling

HEK-293 cells and HK-2 cells were cultured in DMEM medium containing 10% fetal bovine serum (FBS) at 37 °C in an atmosphere of 5% CO_2_ and 95% air in an incubator (HERAcell 150, ThermoFisher Scientific, Waltham, MA, USA). To induce the differentiation of HEK-293 cells and HK-2 cells, the cell concentration was adjusted to 3 × 10^5^ cells/well in 12-well plates, which were then cultured in DMEM medium containing 10% FBS for two days until confluence. HEK-293 cells and HK-2 cells were co-cultured with the medium containing drugs for 24 h, then the cells were lysed, and the contents of each protein in the total extracts were determined. The experiment was divided into four groups for HEK-293 cells: control group (without any treatment), model group (LPS 20 μg/mL), TFSA group (LPS 20 μg/mL + TFSA 50 μg/mL), and M4 group (LPS 20 μg/mL + M4 50 μg/mL). For HK-2 cells, the dose of LPS was 10 μg/mL.

### 4.7. Oxidative Stress and Inflammatory Factors Detection

The detection kits of SOD, GSH, and MDA were purchased from Beyotime Biotechnology. The detection kits of IL-6, TNF-α, IL-1β, TLR4, and NF-κB were purchased from Beijing Solarbio Science & Technology Co., Ltd., Beijing, China. Following the manufacturer’s instructions, the absorbance was measured using a microplate reader (Biotek, Winooski, VT, USA).

### 4.8. Metabolism of PWE and TFSA by Gut Microbiota In Vitro

The in vitro incubation with gut microbiota was performed according to the established method [25]. Reaction systems containing PWE or TFSA (10 μg/mL) were incubated at 37 °C with shaking (200 rpm), with heat-inactivated bacterial cultures serving as the negative control. Aliquots were collected at specified time points (0, 10, 20, 30, 45, 60, 120, 240, 360, 480, 720, and 1440 min). Each sample was immediately quenched with a 3-fold volume of methanol containing glipizide (100 ng/mL) as an internal standard, vortexed for 30 s, and centrifuged at 15,000 rpm for 15 min (4 °C). Subsequently, 3 μL of the supernatant was subjected to LC-MS/MS for quantitative analysis, while another 10 μL aliquot was used for metabolite identification via LC/MS-Q-TOF.

### 4.9. Metabolism of TFSA by Liver Homogenate In Vitro

The in vitro incubation with liver homogenate was performed according to the established method [25]. Reaction systems containing TFSA (10 μg/mL) were incubated at 37 °C with shaking (800 rpm). Aliquots were collected at predetermined time points (0, 15, 30, 45, 60, 90, and 120 min) and the reaction was terminated by adding a 3-fold volume of methanolic glipizide solution (100 ng/mL). Following centrifugation at 15,000 rpm for 15 min (4 °C), 3 μL of the resulting supernatant was analyzed by LC-MS/MS.

### 4.10. Molecular Docking

Docking analysis was conducted using AutoDock vina 1.1.2. The crystal structure of carboxylesterase (1AUO), TNF (2AZ5), TLR4 (2Z62), and NF-κB (1SVC) were obtained from the Protein Data Bank (PDB) database, and the 3D structures of the small molecules of the drug PWE were retrieved and downloaded from PubChem. The docking process of macromolecules and ligands was performed using AutoDock according to default parameters. Each pair of docking was conducted twenty times, and the lowest binding affinity was recorded. The affinity was displayed in a heat map using the GraphPad Prism, and the docking models were visualized by PyMOL 2.5.7 software [25].

Proteins were processed prior to docking to remove water molecules, hydrogenation, and assign Gasteiger charges. The coordinate position of the docking grid center of each protein was determined based on the binding site with the original cocrystal ligand, as detailed in Table S3. Binding affinity, measured in kcal/mol, was used as the primary criterion for assessing binding strength. Lower values indicate more stable binding, and generally values less than -5 kcal/mol are considered good docking.

### 4.11. Effect of BNPP Inhibition on the Transformation of TFSA

The culture system containing TFSA (10 μg/mL) was supplemented with the carboxylesterase inhibitor bis-p-nitrophenyl phosphate (BNPP, 100 μg/mL), following the methodology detailed in Section 4.8. Incubations were then carried out at 37 °C for 0, 2, and 6 h. A parallel culture system without BNPP served as the control.

### 4.12. Potential Ingredients Analysis of PWE

The components of PWE were obtained from Herb (http://herb.ac.cn/, accessed on 16 February 2025) and verified by Swiss Target Prediction (http://www.swisstargetprediction.ch/, accessed on 16 February 2025).

### 4.13. Construction and Analysis of Protein Interaction Network

The relevant targets of drug were obtained from Swiss Target Prediction (http://www.swisstargetprediction.ch/, accessed on 16 February 2025). By adding the keyword “Acute kidney injury” in the GeneCards (https://www.genecards.org/, accessed on 27 August 2025) and OMIM (https://www.omim.org/, accessed on 27 August 2025) databases for retrieval, disease-related targets were obtained. All targets in the database were integrated in Excel, and duplicate genes were removed. The obtained drug component targets and disease targets were mapped to each other, and then a Venn map was made to obtain intersection genes.

The drug-interaction gene was uploaded to the String database (https://string-db.org/, accessed on 18 February 2025) for the construction of PPI networks with the organisms set as ‘Homo sapiens’. The PPI data were imported into the Cytoscape 3.7.2 software for constructing the Drug-Ingredient-Target network. Using the centiscape 2.2 plugin to calculate degree, closeness, and betweenness between nodes. The genes with all three values greater than the median values were defined as the core target. Table S4 systematically lists each core target, describes its known function in the kidney, and provides high-quality literature support (including PMID) for its direct association with AKI.

### 4.14. GO and KEGG Pathway Enrichment Analysis

GO enrichment and KEGG pathway analysis of intersection genes were performed to explore the potential mechanisms and signaling pathways via the ClusterProfiler package in R software 4.1.2 [38]. GO analysis focused on the functional classification of genes, whereas KEGG analysis focused on the role of genes in metabolic pathways. The analysis results with a *p*-value < 0.05 were considered significant. GO enrichment analysis contained 3 components: biological process (BP), cellular component (CC), and molecular function (MF). We select the top 5 BP, CC, MF in the GO function, and 30 pathways related to AKI in KEGG pathway entries (*p* < 0.05) as the main gene function enrichment process.

### 4.15. Target Validation by GEO Datasets

The AKI dataset GSE273063 was utilized to validate the expressions of candidate target genes. Differentially expressed genes (DEGs) were screened by GEO2R with the threshold value |log2 (fold change)| > 1 and *p*-value < 0.05 and displayed in a volcano plot.

### 4.16. S rRNA Sequencing Analysis

Upon completion of the acute kidney injury experiment, fecal samples were collected from the control, model, and PEW-High groups. Microbial DNA was extracted using the E.Z.N.A. Soil DNA Kit (Omega Biotek, Norcross, GA, USA) following the manufacturer’s protocols. The V3-V4 hypervariable region of the 16S rRNA gene was amplified with primers 338F (5′-ACTCCTACGGGAGGCAGCAG-3′) and 806R (5′-GGACTACHVGGGTWTCTAAT-3′). Resulting PCR products were separated on 2% agarose gels and purified with the AxyPrep DNA Gel Extraction Kit (Axygen Biosciences, USA). Finally, the purified amplicons were subjected to Illumina MiSeq sequencing to assess bacterial diversity across the experimental groups.

The PERMANOVA test based on Bray–Curtis distance matrix showed significant differences in bacterial community structure (*p* < 0.05) (Table S5). At the genus level, we analyzed the differential abundance of the top 20 differential microorganisms between Control vs. Model and Model vs. PWEH. Benjamini–Hochberg correction was applied to the *p-values* for all genera to obtain q-values. For the top 20 differential organisms, effect sizes and 95% confidence intervals were calculated (Tables S6 and S7). The above data add statistical rigor and reproducibility.

### 4.17. Metabolomics Analysis of PWE Against AKI

This study was divided into three groups: Control, Model, and PWEH. Each group had *n* = 5, and in total, there were 15 samples. In the sample preparation and mass spectrometry analysis stage, the sequencing order of all samples was completely randomized using a random number table to minimize systematic errors and batch effects to the greatest extent. QC samples composed of equal amounts of all the samples to be tested were prepared. In the instrument analysis sequence, we inserted a QC sample after injecting 5 research samples every time to monitor the stability of the instrument and the repeatability of the data. The median RSD of the QC samples was 11.8%, and 76.5% of the compounds were less than 30%, proving that this method has good stability and repeatability, and the obtained data is reliable (Figure S12A).

An untargeted metabolomics study was performed on LC/MS-Q-TOF (Shimadzu, Japan). Chromatographic separations were performed with an Alltima C18 column (4.6 mm × 150 mm, 5 μm). Water containing 0.1% formic acid (*v*/*v*) was used as mobile phase A and acetonitrile as mobile phase B. The chromatographic column temperature was 40 °C, flow rate 0.4 mL/min, and injection volume 5 μL. The gradient elution program was as follows: 10% B (0–1 min), 10–99% B (1–20 min), 99% B (20–25 min), 99–10% B (25–25.01 min), and 10% B (25.01–30 min). The mass spectrometry setup settings were capillary temperature, 300 °C; heating block temperature, 400 °C; nebulizing gas flow rate, 1.0 L/min; detector voltage, 2.8 kV.

Chromatographic peaks were automatically identified, matched, paired, and normalized using MS-DIAL software 5.5.241113. Metabolites were confirmed with Human Metabolome Database (HMDB, https://hmdb.ca/). *t*-test, principal component analysis (PCA), and orthogonal partial least squares-discriminant analysis (OPLS-DA) were performed using MetaboAnalyst 5.0 (https://www.metaboanalyst.ca/, accessed on 10 June 2025). Significant differential metabolites were identified according to the criteria of variable weight value (VIP) >1 and *p*-value < 0.05. These metabolites were then subjected to clustering, association, and pathway analysis. OPLS-DA analysis was performed on Control vs. Model and Model vs. PWEH to maximize the separation between groups (Figure S12B,C). According to the OPLS-DA model, we identified potential differential metabolites with VIP > 1.0 and *p*-value < 0.05. Subsequently, we conducted a Student’s *t*-test on these metabolites and applied the Benjamini–Hochberg method for FDR correction (Tables S8 and S9). This dataset has been fully made public in the public database MetaboLights, with the accession number: MTBLS13244.

### 4.18. Statistical Analysis

Mass spectrum data acquisition and subsequent data processing were performed with Shimadzu LabSolutions (Kyoto, Japan). One-way ANOVA followed by Dunnett’s multiple comparisons test and two-tailed Student’s *t*-test were used for statistical analysis with GraphPad Prism Version 8 (GraphPad Software, La Jolla, CA, USA). Data are expressed as the means ± standard deviation. *p*-values less than 0.05 were considered statistically significant.

## 5. Conclusions

In this study, a strategy of gut microbiota–drug interaction analysis and network pharmacology was conducted to clarify the nephroprotective effect and potential mechanisms of PWE for the first time. TFSA, the active ingredient of PWE, plays the key role in exerting therapeutic effects, mainly through the biotransformation of microbial carboxylesterase to release active metabolite M4 and subsequently inhibiting the TLR4–NF-κB pathway. In addition, PWE can also improve gut microbiota composition and metabolic disorders. This study provides new insights into the mechanism of PWE participating in renal protection by mediating the gut-kidney axis.

## Figures and Tables

**Table 1:** Quantification ions and optimized MRM parameters of Tenuifoliside A, metabolites and glipizide (IS).

	Analyte	Precursor Ion (m/z)	Quantification	Q1 CE (Volt)	Q2 CE (Volt)	Q3 CE (Volt)	Retention Time (min)
TFSA	Tenuifoliside A	681.25	137.10	20.0	35.0	22.0	7.687
M1	Glomeratose A	561.35	237.15	20.0	20.0	15.0	8.271
M2	4-hydroxybenzoic acid	137.15	92.95	14.0	15.0	16.0	2.365
M3	(E)-3-(3,4,5-trimethoxyphenyl)acrylic acid	237.20	103.15	12.0	16.0	18.0	4.350
M4	3-(3,4,5-trimethoxyphenyl)propanoic acid	239.25	180.10	12.0	14.0	30.0	4.347
IS	Glipizide	444.15	319.15	13.0	22.0	21.0	6.916

## Data Availability

The original contributions presented in this study are included in the article/supplementary material. Further inquiries can be directed to the corresponding author.

## Supplementary Materials

The following supporting information can be downloaded at: https://www.mdpi.com/article/10.3390/ijms262210889/s1. Figure S1: Investigation of potential renal-protection targets of *Polygala tenuifolia* Willd. compounds based on network pharmacology; (A) Venn diagram showing the intersections of *Polygala tenuifolia* Willd. compounds and AKI. (B) *Polygala tenuifolia* Willd. compounds–common target network. (C) PPI network of the core target; Figure S2: Chemical quality control of PWE; Figure S3: The metabolic stability of TFSA in simulated gastric fluid and artificial intestinal juice. (A) Relative abundance of TFSA in PWE in the co-incubation system of simulated gastric fluid (*n* = 3). (B) Relative abundance of TFSA in PWE in the co-incubation system of artificial intestinal juice (*n* = 3). (C) In vitro metabolic curve of TFSA in the simulated gastric fluid (*n* = 3). (D) In vitro metabolic curve of TFSA in the artificial intestinal juice (*n* = 3). (E) The abundance of TFSA in the liver homogenate (*n* = 3). (F) The extract ion chromatogram of TFSA, M1, M2, M3, M4, and glipizide (IS). Data are presented as mean ± SD, and a two-tailed Student’s *t*-test was used for analysis (*** *p* < 0.001, ** *p* < 0.01, * *p* < 0.05); Figure S4: The MS/MS data and the mass spectrometric cleavage pathway of M1. (A) The extracted ion chromatograms of M1 for 0, 1, 8, and 12h. (B) The MS/MS data of M1. (C) The possible structure of M1 and the mass spectrometric cleavage pathway; Figure S5: The MS/MS data and the mass spectrometric cleavage pathway of M3. (A) The extracted ion chromatograms of M3 for 0, 1, 8 and 12h. (B) The MS/MS data of M3. (C) The possible structure of M3 and the mass spectrometric cleavage pathway; Figure S6: The MS/MS data and the mass spectrometric cleavage pathway of M4. (A) The extracted ion chromatograms of M4 for 0, 1, 8, and 12h. (B) The MS/MS data of M4. (C) The possible structure of M4 and the mass spectrometric cleavage pathway; Figure S7: Depletion verification of PGF status. (A) Representative images of aerobic (left) and anaerobic (right) bacteria cultured in fresh feces of Control and ABX mice. (B) Number of colonies of aerobic (left) and anaerobic (right) bacteria in fresh feces of Control and ABX mice (*n* = 3). Data are presented as mean ± SD, and a two-tailed Student’s *t*-test was used for analysis (*** *p* < 0.001); Figure S8: Distribution of TFSA and its metabolites in feces and kidneys of normal and PGF mice. (A) The content of TFSA, M1, M3, and M4 in the feces of normal and PGF mice after oral administration of TFSA (*n* = 5, 30 mg/kg). (B) The content of TFSA and M4 in the kidneys of normal and PGF mice after oral administration of TFSA (*n* = 5, 30 mg/kg). Data are presented as mean ± SD, and a two-tailed Student’s *t*-test was used for analysis (*** *p* < 0.001, ** *p* < 0.01); Figure S9: Investigation of the potential targets of TFSA and its metabolites based on network pharmacology. (A) Venn diagram showing the intersections of TFSA and its metabolites, and AKI. (B) TFSA–metabolites–common target network; Figure S10: Visualization of NF-κB signaling pathways; Figure S11: TFSA and M4 inhibited the phosphorylation of NF-κB in HK-2 cell. (A) Calibration of LPS dosage *(n* = 4). (B) WB detection of protein expression of NF-κB P65; P-NF-κB P65 in total protein (*n* = 3). Data are presented as mean ± SD, and statistical analysis was performed with one-way ANOVA followed by Dunnett's multiple comparisons test (*** *p* < 0.001, ** *p* < 0.01, * *p* < 0.05); Figure S12: Model analysis of the effectiveness and reliability of metabolomics. (A) Relative standard deviation (RSD) distribution map. (B) OPLS-DA scores of samples in Control vs. Model. (C) OPLS-DA scores of samples in Model vs. PWEH; Table S1: Characteristics of Tenuifoliside A in the gut microbiota by LC/MS-Q-TOF; Table S2: Characteristics of M1, M3, and M4 in gut microbiota by LC/MS-Q-TOF; Table S3: Molecular docking parameters; Table S4: Relationship between the target and renal phenotype; Table S5:The results of the PERMANOVA test; Table S6: The microbiome analysis of Control vs. Model; Table S7: The microbiome analysis of Model vs. PWEH; Table S8: The differential metabolites of Control vs. Model; Table S9: The differential metabolites of Model vs. PWEH.

## Abbreviations

The following abbreviations are used in this manuscript:

AKI	Acute kidney injury
BNPP	bis-p-nitrophenyl phosphate
BP	Biological process
CC	Cellular component
CCK8	Cell counting kit 8
CE	Carboxylesterase
DMEM	Dulbecco’s Modified Eagle’s medium
EICs	Extracted ion chromatograms
ELISA	Enzyme-linked immunosorbent assay
FBS	Fetal bovine serum
GC	Gas chromatography
GO	Gene Ontology
HEK-293 cells	Human embryonic kidney 293 cells
IL-1β	Interleukin-1β
IL-6	Interleukin-6
IS	Internal standard
KEGG	Kyoto Encyclopedia of Genes and Genomes
LC–MS/MS	Liquid chromatography tandem mass spectrometry
LC/MS–Q–TOF	Liquid chromatography tandem mass spectrometry-quadrupole/time of flight mass spectrometer
MF	Molecular function
MRM	Multiple reaction monitoring
NF-κB	Nuclear factor kappa-B
PGF	*Pseudo*-germ-free
PPI	Protein interaction network
PWE	*Polygala tenuifolia* Willd. extracts
SCFAs	Short–chain fatty acids
SD	Sprague–Dawley
TCM	Traditional Chinese medicine
TFSA	Tenuifoliside A
TLR4	Toll-like receptor 4
TNF-α	Tumor necrosis factor-α
