# Supplementary material for: Integrated Gut Microbiota–Drug Interaction Analysis and Network Pharmacology for the Investigation of Renal-Protective Effect of Polygala tenuifolia Willd
Source: Int J Mol Sci. 2025 Nov 10;26(22):10889. doi: 10.3390/ijms262210889 (PMC12652610; doi:10.3390/ijms262210889)
Supplement: Supplementary file 1 [file ijms-26-10889-s001.zip › ijms-3929635-supplementary.pdf]

Supplementary information

Integrated Gut Microbiota–Drug Interaction Analysis and Network Pharmacology for the Investigation of Renal-Protective Effect of *Polygala tenuifolia* Willd

Jia-Chun Hu †, Jian-Ye Song †, Ru Feng, Meng-Liang Ye, Hui Xu, Jin-Yue Lu, Heng-Tong Zuo, Yi Zhao, Jing-Yue Wang, Jing-Yu Jin, Ling-Yu Wei, Yong-Mei Tu and Yan Wang *

State Key Laboratory of Bioactive Substance and Function of Natural Medicines, Institute of Materia Medica, Chinese Academy of Medical Sciences/Peking Union Medical College, Beijing 100050, China;
hujiachun@imm.ac.cn (J.-C.H.); songjianye@imm.ac.cn (J.-Y.S.); fengru@imm.ac.cn (R.F.);
yemengliang@imm.ac.cn (M.-L.Y.); xuhui@imm.ac.cn (H.X.); lujinyue@imm.ac.cn (J.-Y.L.);
zuohengtong@imm.ac.cn (H.-T.Z.); zhaoyi@imm.ac.cn (Y.Z.); wangjingyue12@126.com (J.-Y.W.);
jennyjin7@163.com (J.-Y.J.); 13653440838@163.com (L.-Y.W.); 18783106853@163.com (Y.-M.T.);
* Correspondence: wangyan@imm.ac.cn; Tel.: +86-10-6316-5238
† These authors contributed equally to this work.

Supplementary material includes:

1. Supplementary Figures and their legends 1–12.
2. Supplementary Table 1–9.

Supplementary Figures and Their Legends

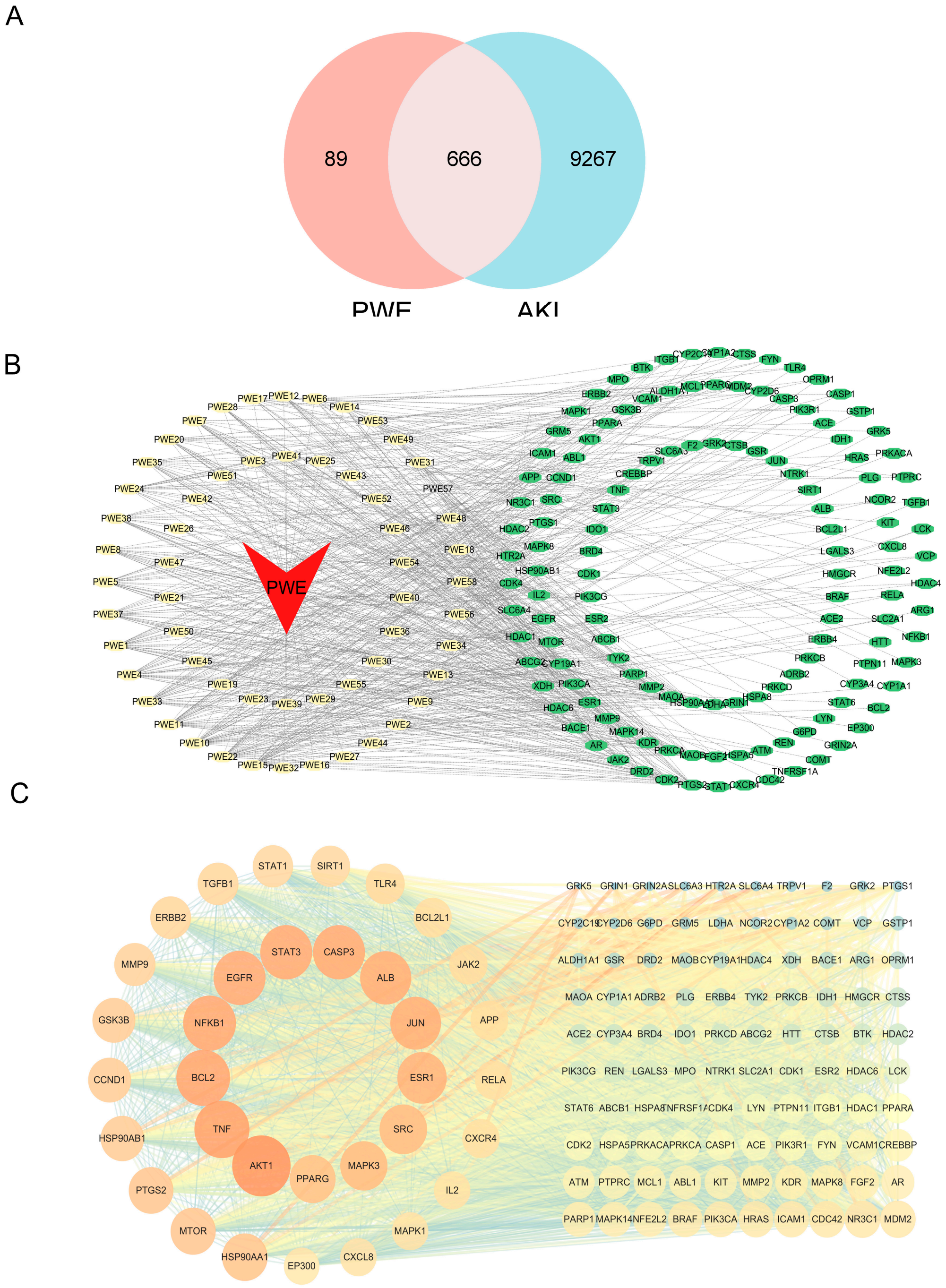

Supplementary Figure S1. Investigation of potential renal-protection targets of *Polygala tenuifolia* Willd. compounds based on network pharmacology. (A) Venn diagram showing the intersections of *Polygala tenuifolia* Willd. compounds and AKI. (B) *Polygala tenuifolia* Willd. compounds–common target network. (C) PPI network of the core target.

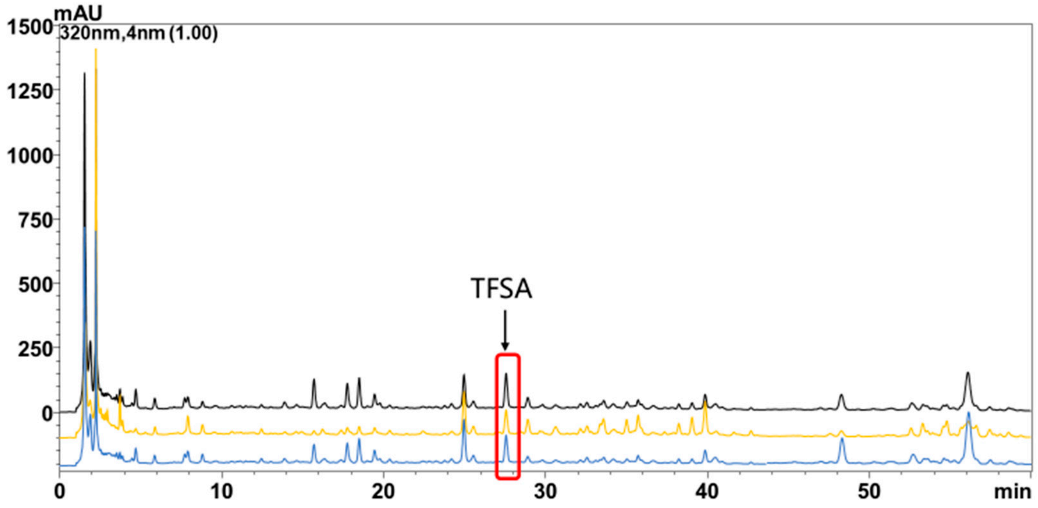

Supplementary Figure S2. Chemical quality control of PWE.

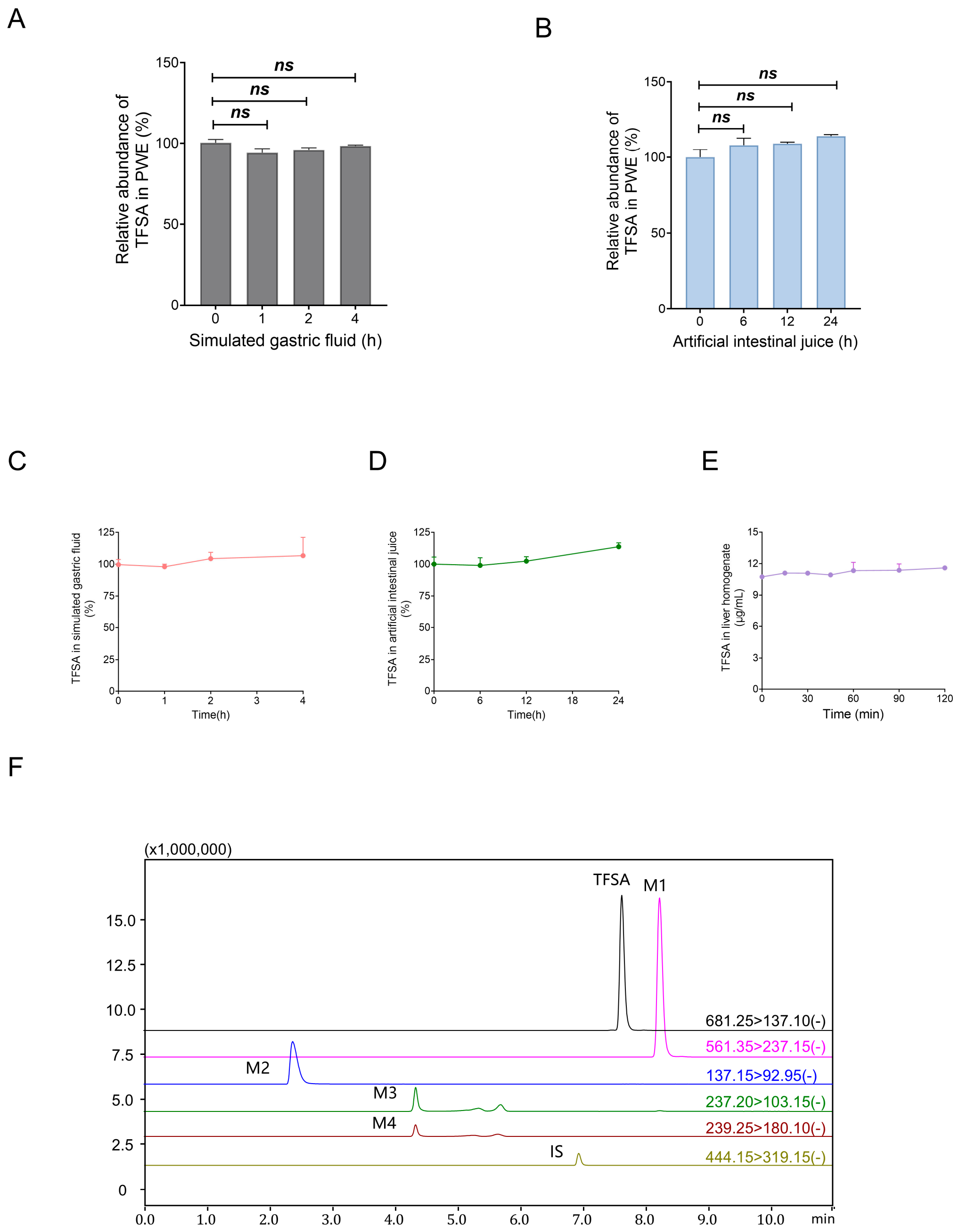

Supplementary Figure S3. The metabolic stability of TFSA in simulated gastric fluid and artificial intestinal juice. (A) Relative abundance of TFSA in PWE in the co-incubation system of simulated gastric fluid (n = 3). (B) Relative abundance of TFSA in PWE in the co-incubation system of artificial intestinal juice (n = 3). (C) In vitro metabolic curve of TFSA in the simulated gastric fluid (n = 3). (D) In vitro metabolic curve of TFSA in the artificial intestinal juice (n = 3). (E) The abundance of TFSA in the liver homogenate (n = 3). (F) The extract ion chromatogram of TFSA, M1, M2, M3, M4, and glipizide (IS). Data are presented as mean ± SD, and a two-tailed Student's t test was used for analysis (***$P < 0.001$, **$P < 0.01$, *$P < 0.05$).

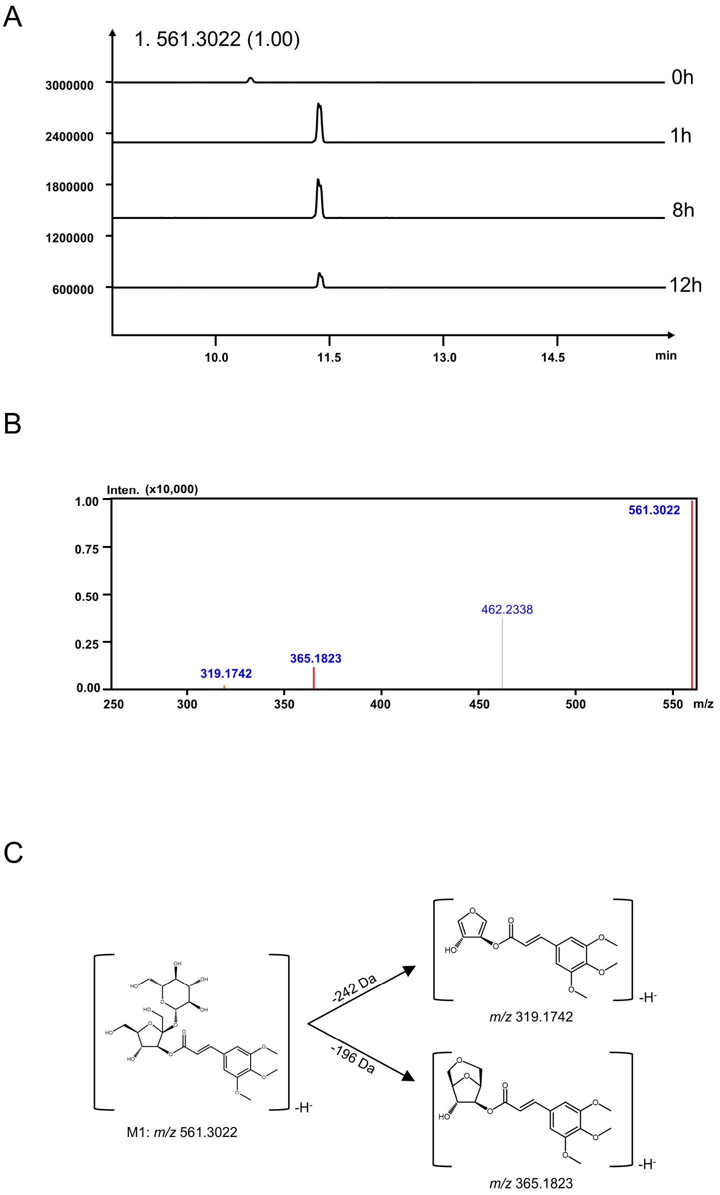

Supplementary Figure S4. The MS/MS data and the mass spectrometric cleavage pathway of M1. (A) The extracted ion chromatograms of M1 for 0, 1, 8, and 12h. (B) The MS/MS data of M1. (C) The possible structure of M1 and the mass spectrometric cleavage pathway.

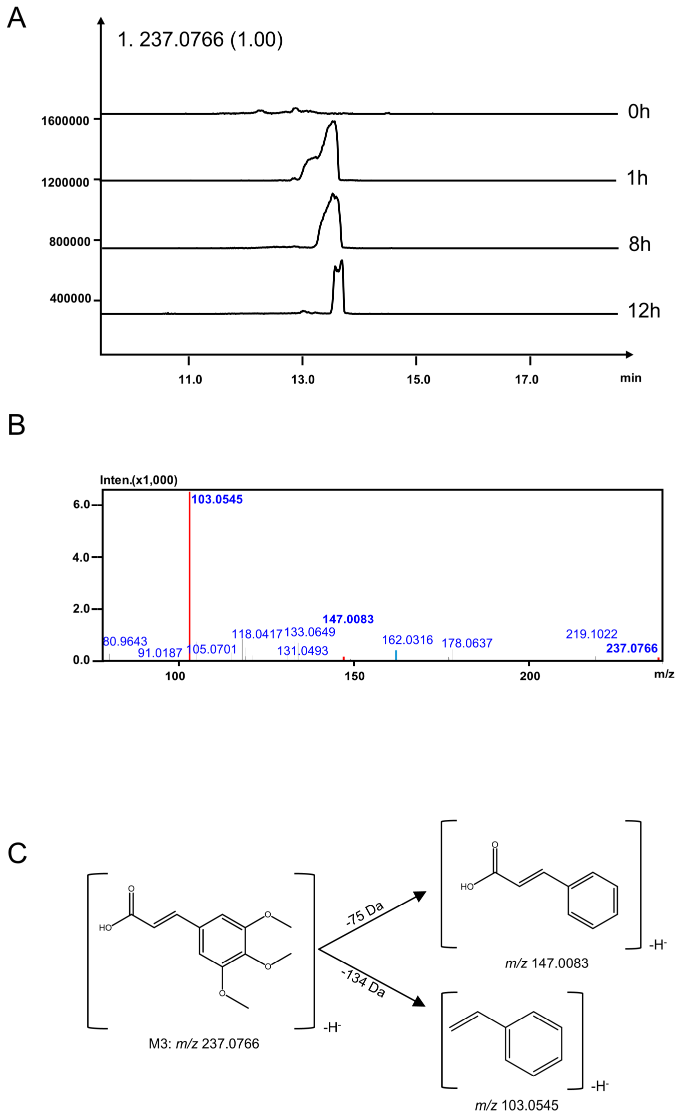

Supplementary Figure S5. The MS/MS data and the mass spectrometric cleavage pathway of M3. (A) The extracted ion chromatograms of M3 for 0, 1, 8 and 12h. (B) The MS/MS data of M3. (C) The possible structure of M3 and the mass spectrometric cleavage pathway.

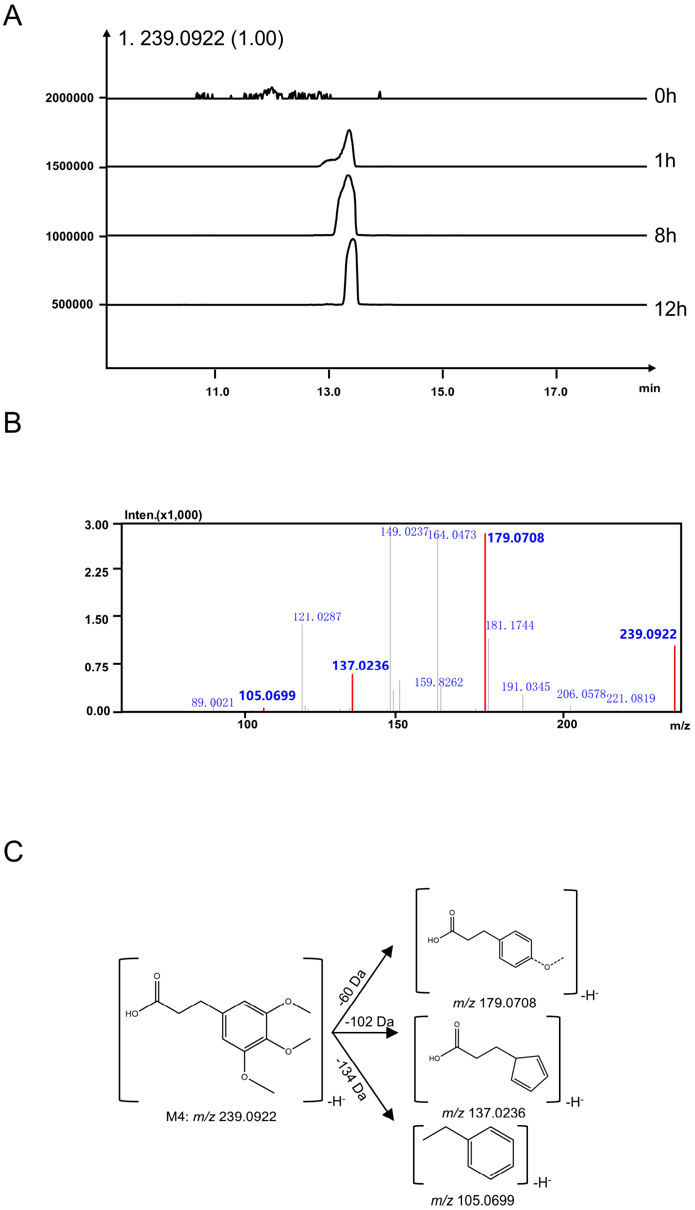

Supplementary Figure S6. The MS/MS data and the mass spectrometric cleavage pathway of M4. (A) The extracted ion chromatograms of M4 for 0, 1, 8, and 12h. (B) The MS/MS data of M4. (C) The possible structure of M4 and the mass spectrometric cleavage pathway.

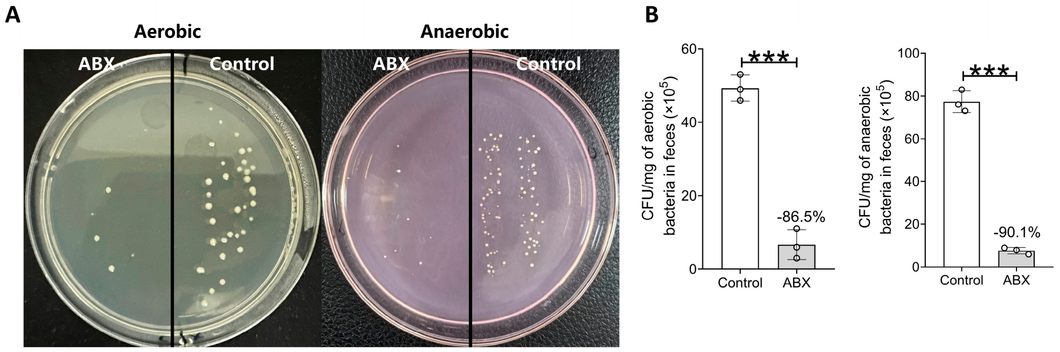

Supplementary Figure S7. Depletion verification of PGF status. (A) Representative images of aerobic (left) and anaerobic (right) bacteria cultured in fresh feces of Control and ABX mice. (B) Number of colonies of aerobic (left) and anaerobic (right) bacteria in fresh feces of Control and ABX mice (*n* = 3). Data are presented as mean ± SD, and a two-tailed Student's t test was used for analysis (****P* < 0.001).

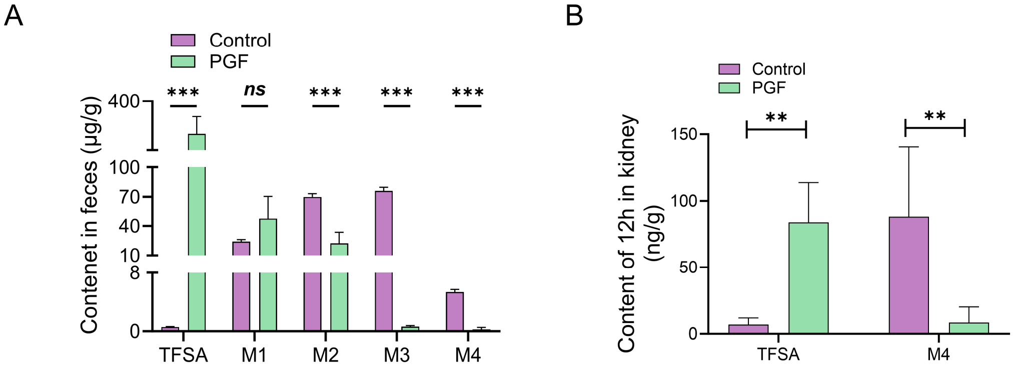

Supplementary Figure S8. Distribution of TFSA and its metabolites in feces and kidneys of normal and PGF mice. (A) The content of TFSA, M1, M3, and M4 in the feces of normal and PGF mice after oral administration of TFSA (*n* = 5, 30 mg/kg). (B) The content of TFSA and M4 in the kidneys of normal and PGF mice after oral administration of TFSA (*n* = 5, 30 mg/kg). Data are presented as mean ± SD, and a two-tailed Student's t test was used for analysis (****P* < 0.001, ***P* < 0.01).

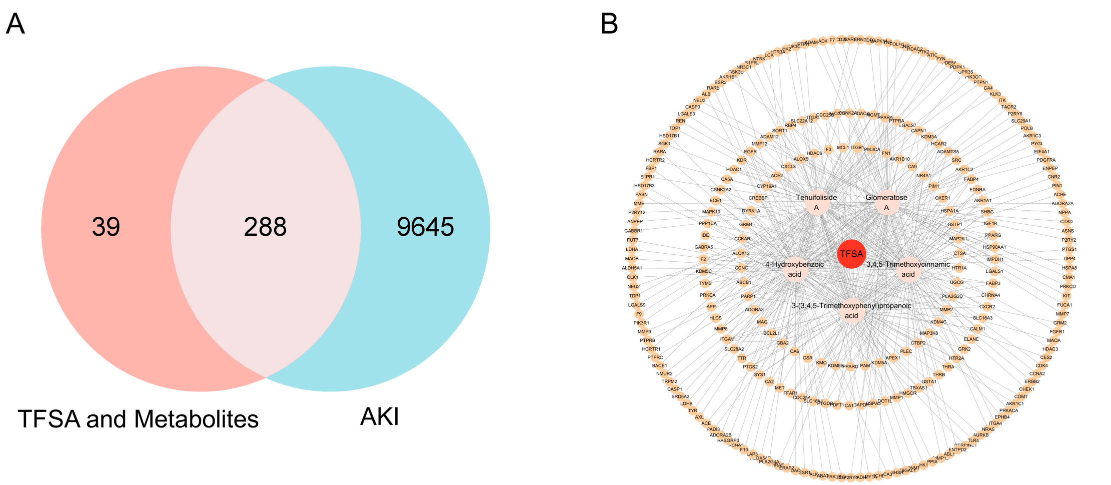

Supplementary Figure S9. Investigation of the potential targets of TFSA and its metabolites based on network pharmacology. (A) Venn diagram showing the intersections of TFSA and its metabolites, and AKI. (B) TFSA–metabolites–common target network.

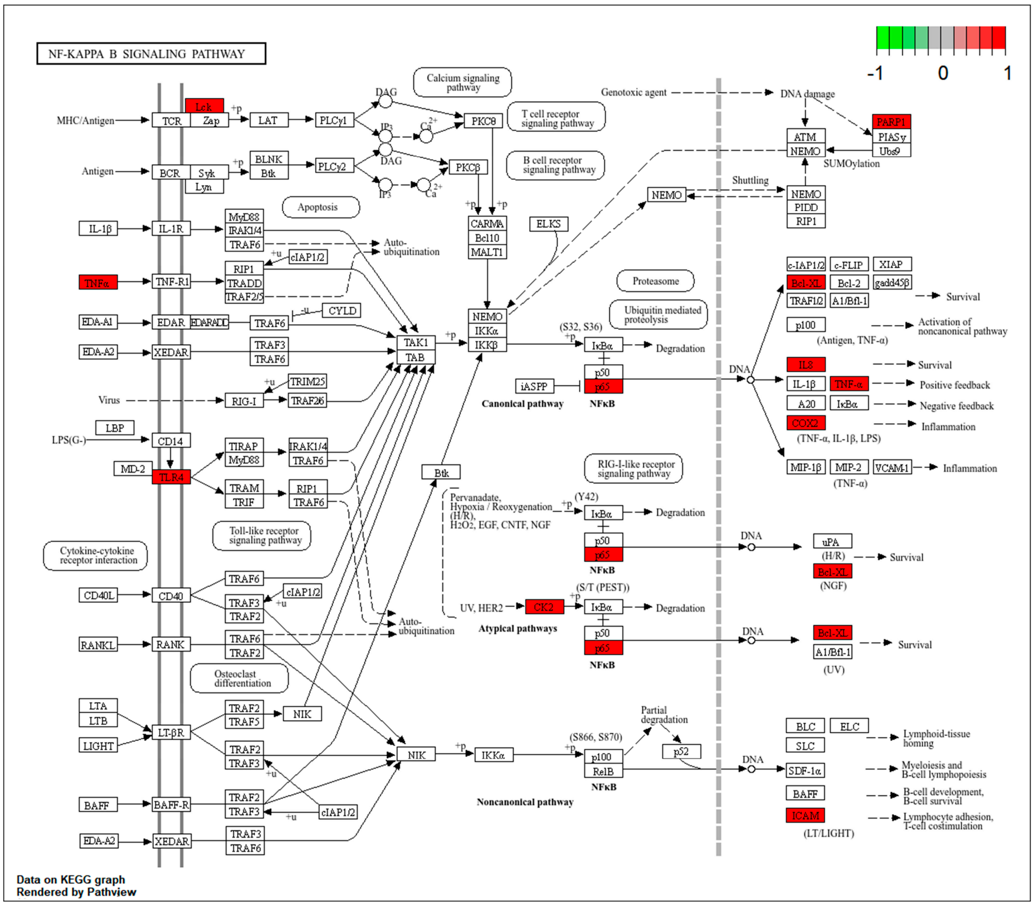

Supplementary Figure S10. Visualization of NF-κB signaling pathways.

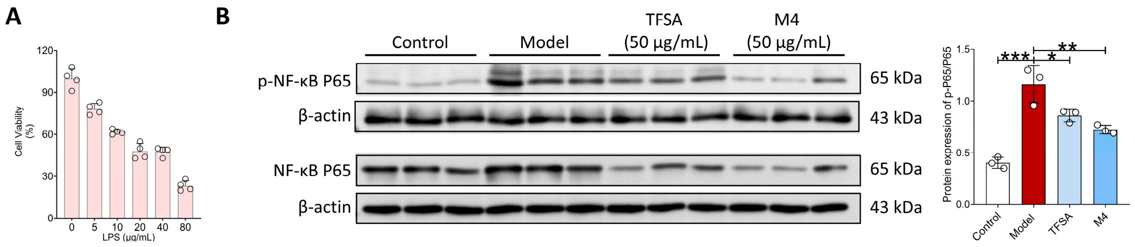

Supplementary Figure S11. TFSA and M4 inhibited the phosphorylation of NF-κB in HK-2 cell. (A) Calibration of LPS dosage (n = 4). (B) WB detection of protein expression of NF-κB P65; P-NF-κB P65 in total protein (n = 3). Data are presented as mean ± SD, and statistical analysis was performed with one-way ANOVA followed by Dunnett's multiple comparisons test (***P < 0.001, **P < 0.01, *P < 0.05).

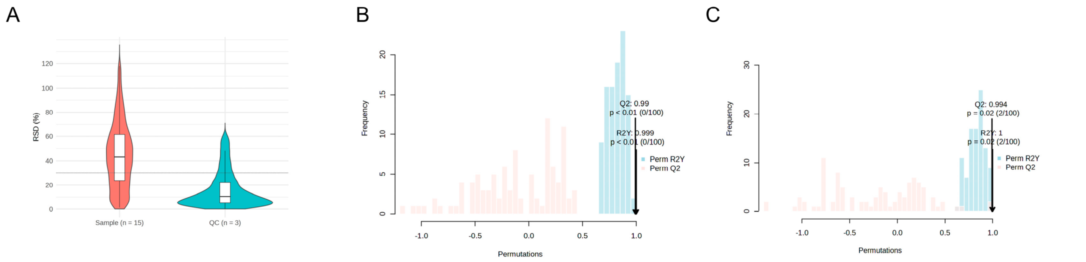

Supplementary Figure S12. Model analysis of the effectiveness and reliability of metabolomics. (A) Relative standard deviation (RSD) distribution map. (B) OPLS-DA scores of samples in Control vs. Model. (C) OPLS-DA scores of samples in Model vs. PWEH.

Supplementary Table 1. Characteristics of Tenuifoliside A in the gut microbiota by LC/MS-Q-TOF.

Compound name	Retention time (min)	Predicted molecular weight	Molecular formula	Fragment characteristics	
				Mass spectrometry (MS;$(M\text{-}H]^-$)	MS/MS
Tenuifoliside A	14.226	681.2036	$C_{31}H_{38}O_{17}$	681.20146	443.11769, 239.05456 and 137.02315

Supplementary Table 2. Characteristics of M1, M3, and M4 in gut microbiota by LC/MS-Q-TOF.

Metabolites	Retention time (min)	Reaction	Predicted molecular weight	Molecular formula	Fragment characteristics	
					Mass spectrometry (MS;$(M\text{-}H]^-$)	MS/MS
M1	11.3	Esterolysis	562.19	$C_{24}H_{34}O_{15}$	561.30	319.17 and 365.18
M3	13.5	Esterolysis	238.08	$C_{12}H_{14}O_5$	237.08	147.01 and 103.05
M4	13.6	Esterolysis and reduction	240.10	$C_{12}H_{16}O_5$	239.09	179.07, 137.02 and 105.07

Supplementary Table 3. Molecular docking parameters.

Name	Docking grid center coordinates(x,y,z)	Grid size(x×y×z)
Carboxylesterase (1AUO)	(45.075,30.699,59.055)	40×44×40
TNF (2AZ5)	(-13.63,71.606,27.002)	126×126×126
TLR4 (2Z62)	(18.328,0.115,8.276)	126×126×96
NF-κB (1SVC)	(42.381,14.683,38.036)	126×126×96

Supplementary Table 4. Relationship between the target and renal phenotype.

Target	Function	PMID
TNF	Inflammation、Immunity	37949330
TLR4	Inflammation	39399126
NF-κB	Inflammation	37284446

Supplementary Table 5. The results of the PERMANOVA test.

Group	Permutations	pseudo-F	R2	p-value
All	999	5.9114	0.6634	0.005

Supplementary Table 6. The microbiome analysis of Control vs. Model.

Taxon	p.value	q.value	FDR	Cohen's d	CI_lower	CI_upper
g_[Prevotella]	0.428228144	0.541769569	0.012784199	0.827591674	-1.534414773	3.189598122
g_[Ruminococcus]	0.366209447	0.541769569	0.007405977	-0.496550953	-2.798178012	1.805076107
g_Adlercreutzia	0.491975321	0.606056555	0.031840348	-0.120871479	-2.389898474	2.148155516
g_AF12	0.643668044	0.712307882	0.008868746	-0.561884229	-2.873141217	1.74937276
g_Akkermansia	0.40289592	0.541769569	0.003431795	0.77151675	-1.578264445	3.121297945
g_Bacteroides	0.656551192	0.715472453	0.908368967	0.173595329	-2.097628315	2.444818973
g_Blautia	-	-	-	-	-	-
g_Butyricimonas	0.005852304	0.075924564	0.011704607	-2.016998707	-4.80133203	0.767334617
g_Coprococcus	0.72853429	0.783866008	0.124906853	0.475744553	-1.823057667	2.774546773
g_Dehalobacterium	0.004995885	0.075924564	0.001224039	-3.074732822	-6.423197555	0.273731911
g_Enterococcus	0.040366206	0.190618194	0.641850864	-1.396465084	-3.924671402	1.131741235
g_Odoribacter	0.021685942	0.142402681	0.012256163	-2.445125428	-5.441740108	0.551489253
g_Oscillospira	0.016021486	0.142245104	0.002016448	-1.632466865	-4.249913528	0.984979798

g_Parabacteroides	0.021779234	0.142402681	0.178293943	-1.778582776	-4.45649211	0.899326557
g_Prevotella	0.292548732	0.541769569	0.087872108	1.415191704	-1.119695019	3.950078427
g_Proteus	0.028991175	0.164283325	0.089594676	-1.143686875	-3.588958534	1.301584783
g_Ruminococcus	0.033303847	0.176926688	0.017317115	-1.594189061	-4.196435442	1.00805732
g_Staphylococcus_f_Staphylococcaceae	0.242527783	0.541769569	0.004539873	-0.375127923	-2.661936974	1.911681128
g_Sutterella	0.261086451	0.541769569	0.031387966	0.917890293	-1.465452754	3.30123334
g_Trabulsiella	-	-	-	-	-	-

Supplementary Table 7. The microbiome analysis of Model vs. PWEH.

Taxon	p.value	q.value	FDR	Cohen's d	CI_lower	CI_upper
g_[Prevotella]	0.007031309	0.037969071	0.525470679	1.151503557	-1.296123541	3.599130654
g_[Ruminococcus]	0.002592092	0.024004166	0.112274734	1.958436265	-0.798909762	4.715782293
g_Adlercreutzia	0.022288244	0.07849338	0.004903811	4.634640611	0.282915802	8.986365421
g_AF12	0.003547498	0.026122488	0.044091865	1.703961097	-0.942594988	4.350517183
g_Akkermansia	0.000686359	0.013898769	-1.780105402	-1.782672273	-4.462327841	0.896983294
g_Bacteroides	0.908368967	0.970068842	8.74109767	0.141657659	-2.128141671	2.411456989

g_Blautia	0.002227735	0.024004166	-0.453800795	-3.067005201	-6.410914247	0.276903845
g_Butyricimonas	0.005852304	0.033859757	0.104070092	2.016998707	-0.767334617	4.80133203
g_Coprococcus	0.106170825	0.307137031	-0.122127724	-1.225419084	-3.695993995	1.245155826
g_Dehalobacterium	0.000122404	0.004957358	0.002951551	6.068056889	0.702179283	11.43393449
g_Enterococcus	0.609758321	0.695639774	-0.21649777	-0.592410751	-2.908559369	1.723737867
g_Odoribacter	0.005515273	0.033859757	0.019601622	2.962455831	-0.3203451	6.245256762
g_Oscillospira	0.000100822	0.004957358	0.373214795	2.586969395	-0.485198861	5.65913765
g_Parabacteroides	0.160464549	0.433254281	-1.672673663	-0.739985418	-3.083242915	1.603272078
g_Prevotella	0.065904081	0.20531656	0.045401723	1.077107962	-1.348664352	3.502880275
g_Proteus	0.071675741	0.215027223	0.19617454	1.093777676	-1.33678153	3.524336883
g_Ruminococcus	0.010390269	0.047189538	0.058360123	2.161751163	-0.691506982	5.015009309
g_Staphylococcus_f_Staphylococcaceae	0.001134968	0.015322071	0.003300352	11.95914412	2.109572937	21.8087153
g_Sutterella	0.020402178	0.07511711	-0.044000584	-2.62584463	-5.719127218	0.467437958
g_Trabulsiella	0.00067257	0.013898769	-0.065294409	-2.884875977	-6.122988468	0.353236514

Supplementary Table 8. The differential metabolites of Control vs. Model.

name	log2(FC)	p.value	Vip	FDR
L-ALANINE	-2.5643	0.00013616	1.272799916	0.00064299
Choline	1.303	4.38E-06	1.328460948	4.3e-05
Creatinine	-4.7015	5.85E-06	1.312667205	5.6338e-05
indole	4.0359	0.0012822	1.19977011	0.0038882
PHENYLETHANOLAMINE	-0.70562	0.00014738	1.284967091	0.00068959
Urea	-4.5058	2.19E-08	1.347801265	5.2877e-07
L-Pyroglutamic acid; CE0; ODHCTXKNWHHXJC-VKHMYHEASA-N	1.1963	0.00022067	1.261787942	0.00096601
N-Methyl-L-proline; CE0; CWLQUGTUXBXTLF-YFKPBYRVSA-N	-1.7872	1.54E-07	1.34583946	2.5707e-06
Creatine	-2.2785	1.01E-07	1.343764346	1.8676e-06
Thiazolidine-4-carboxylic acid	-3.2855	3.10E-05	1.306210023	0.00020369
2-Aminobenzoic acid; CE0; RWZYAGGXGHYGMB-UHFFFAOYSA-N	-3.2867	0.0026766	1.193442855	0.0071096
N-METHYLGLUTAMATE	5.9033	1.79E-06	1.338493936	2.0796e-05
1-Naphthylamine	3.4504	1.87E-06	1.326009192	2.1177e-05
Proline betaine; CE0; CMUNUTVVOOHQPW-LURJTMIESA-N	-2.2588	6.41E-07	1.345193488	8.8321e-06
Indole-3-carboxyaldehyde	3.1877	8.39E-06	1.31333396	7.6298e-05
SPERMIDINE	-1.6063	0.00017679	1.263291893	0.00078403
TAURINE	-1.6474	1.84E-06	1.324590731	2.1074e-05
L-Glutamic acid; CE0; WHUUTDBJXJRKMK-VKHMYHEASA-N	-0.69455	3.87E-05	1.301655438	0.00023343

Methionine	0.858	0.00020216	1.255294504	0.00089266
D-Xylulose	-6.0611	3.06E-05	1.295109835	0.0002029
Vanillin	-4.1554	6.53E-09	1.353364838	1.8005e-07
4-Aminosalicylic acid; CE0; WUBBRNOQWQTFEX-UHFFFAOYSA-N	-5.1427	2.87E-05	1.315197425	0.00019364
UREIDOPROPIONATE	-3.9554	1.72E-05	1.31042694	0.00013208
Allantoin; CE0; POJWUDADGALRAB-UHFFFAOYSA-N	-1.8906	3.45E-05	1.309886718	0.000216
1-Benzylimidazole	4.8011	0.00012607	1.273398467	0.00059808
PHENYLALANINE	-0.28479	0.0049467	1.140132102	0.011844
Uric acid; CE10; LEHOTFFKMJEONL-UHFFFAOYSA-N	2.704	7.14E-05	1.287294547	0.0003793
3-METHYL-2-OXINDOLE	2.7805	0.00090538	1.219462367	0.002979
ADENINE	2.1306	0.00038401	1.235209473	0.001467
1-Methyl-4-phenyl-1,2,3,6-tetrahydropyridine	2.3629	0.01691	1.054796844	0.033491
ARABITOL	-2.8497	6.77E-05	1.291358353	0.00036845
2,4-Toluene diisocyanate	-1.9298	5.24E-05	1.295085358	0.00030187
P-OCTOPAMINE	1.7377	0.00067917	1.219684203	0.0023404
Herniarin	-5.2887	7.65E-11	1.351733474	7.9564e-09
Allantoic acid	0.87346	0.00072526	1.23847147	0.0024824
Serotonin	0.37217	0.0067149	1.098097146	0.015566
HISTIDINE	-0.77174	7.29E-06	1.322372022	6.7554e-05

Diallyl Trisulfide	3.9792	0.0010989	1.210541664	0.0034382
HIPPURATE	-3.2686	1.05E-05	1.313673034	9.0756e-05
Tyrosine	1.6321	1.16E-05	1.313344553	9.6622e-05
4-PYRIDOXATE	-5.2718	3.35E-05	1.301779137	0.00021364
Selegiline	1.4695	0.0014911	1.212357764	0.0043972
THIOPURINE S-METHYLETHER	4.6756	3.38E-06	1.328182452	3.4879e-05
Kynurenic acid	-6.4206	5.99E-08	1.344864299	1.222e-06
4-Nitroquinoline-1-oxide	1.5168	0.00042839	1.256556176	0.0016124
Clopyralid	0.76783	0.0073549	1.104520253	0.016858
5-Hydroxy-3-indoleacetic acid	-3.7367	0.0015369	1.207397062	0.0045046
Phenylacetylglycine	-6.7071	1.07E-08	1.347064881	2.7257e-07
3,4-DIHYDROXY-L-PHENYLALANINE	-2.4193	0.005816	1.103692142	0.013764
N-FORMYL-L-METHIONINE	-0.52226	0.0087107	1.077022774	0.019484
Cysteine-S-sulfate; CE0; NOKPBJYHPHHWAN-REOHCLBHSA-N	-1.7045	2.47E-05	1.300128502	0.00017721
RICININE	-0.71999	0.0112007	1.068236813	0.023915
GALACTOSE	3.6223	2.38E-08	1.35288935	5.2877e-07
Tryptophan	-0.56616	0.02713	1.000311715	0.049254
Phenylethylmalonamide	-6.855	3.43E-05	1.305017864	0.000216
N-ACETYL-L-PHENYLALANINE	-6.1378	1.53E-08	1.348315305	3.8131e-07
9,10-Phenanthrenedione	-4.3254	5.85E-05	1.28270594	0.00032944

1,1,2-Trimethyl-1H-benzo[e]indole	3.5906	0.00064378	1.233287962	0.0022565
N-Acetylglutamine	-3.3658	1.35E-06	1.329723379	1.6173e-05
Aconitic Acid	3.2531	0.0040817	1.163757032	0.010179
Juglone	3.6016	2.35E-08	1.343758276	5.2877e-07
Citrulline	-1.8713	1.33E-06	1.337148519	1.6124e-05
D-Pantothenic Acid	-3.3478	4.26E-06	1.328522199	4.2222e-05
4-O-Methylphloracetophenone	1.3238	2.71E-05	1.307538493	0.00018877
alpha-oxo-1h-indole-3-propanoic acid	6.7878	1.44E-05	1.312525909	0.00011226
Citric acid	3.9634	1.74E-10	1.352084487	1.2704e-08
BUTYL PARABEN	-4.9863	7.97E-11	1.350482622	7.9564e-09
Felbamate	-2.5905	1.27E-07	1.344843503	2.159e-06
L-Cystine	-1.8559	7.50E-07	1.335911239	9.8075e-06
L-Anserine; CE0; MYYIAHXIVFADCU-QMMMGPOBSA-N	-0.88925	0.00065222	1.210410485	0.0022628
Uridine; CE0; DRTQHJPVMGBUCF-XVFCMESISA-N	-2.4267	1.05E-07	1.347154262	1.9196e-06
Patchouli alcohol	2.1135	1.66E-05	1.313706184	0.0001279
N1-Acetylspermine	1.0033	0.010824	1.067616501	0.023145
Pinacidil	-0.52084	0.023128	1.014165767	0.043606
Isoleucylaspartate	1.222	0.0089267	1.088437142	0.019798
Genipin	3.6722	5.72E-07	1.340738968	7.9917e-06
Kainic Acid	-4.8416	3.18E-05	1.311498513	0.00020802

Chrysin	-4.3496	3.04E-09	1.351089875	1.0326e-07
sn-Glycero-3-phosphocholine	2.1043	4.16E-05	1.302992973	0.00024955
Diaveridine	0.95848	0.0024354	1.192358116	0.0066421
falcarindiol	-0.84073	0.00065455	1.235244102	0.0022632
Methohexital	-2.2338	8.95E-05	1.292743358	0.00044739
Alnustone	-1.4616	0.0037141	1.136561984	0.0093671
Vitamin B1	-0.82559	0.0047326	1.120240663	0.011439
Magnolol; PlaSMA ID-1190	-6.2501	3.25E-05	1.30438068	0.0002098
Azacyclonol	-1.657	0.0002439	1.26475624	0.0010241
ellipticine	-5.3341	6.85E-05	1.287833401	0.00036845
Daidzein	-6.7395	1.86E-11	1.353554267	2.7075e-09
Linolenic acid	0.85772	0.00031225	1.239718981	0.0012441
Linoleic acid	1.0181	0.0095375	1.079386175	0.020921
Diazepam	-3.0684	3.74E-06	1.330714714	3.8145e-05
XANTHOSINE	4.6017	7.74E-05	1.290407548	0.00040303
Diazoxon	5.1051	7.50E-12	1.353244757	1.5304e-09
Thiamethoxam	-2.4921	0.00026553	1.267558609	0.0010946
6-PHOSPHOGLUCONATE	-5.051	1.19E-06	1.333651649	1.4647e-05
Retinoic acid	-4.8071	0.0022397	1.179285455	0.0061744
Hexaethylene glycol	1.7955	2.74E-05	1.317272995	0.00018877
(9Z)-9-octadecenoic acid	0.89572	0.019981	1.013920892	0.038382

Arachidonic Acid	0.89277	0.018674	1.021972958	0.036559
Fructoselysine	-0.85307	0.0015442	1.204361352	0.0045132
gamma-Glutamyltyrosine	2.4626	0.0018266	1.180740816	0.0052043
Loureirin B	0.25404	0.0044862	1.124348638	0.010895
steviol	-3.1584	0.015855	1.046785353	0.031727
alpha-Linolenoyl Ethanolamide	1.2584	0.015688	1.033104177	0.031561
Chloramphenicol	-5.2208	3.25E-05	1.293364293	0.0002098
Valaciclovir	-1.5916	0.00015198	1.274345493	0.00070143
Sulcotrione	3.118	1.44E-05	1.317027344	0.00011226
xylan	4.6102	7.04E-05	1.293036324	0.000376
Aflatoxin G1	-4.5991	9.35E-09	1.345261534	2.4466e-07
Tulipinolide	-1.0696	0.00096182	1.219649574	0.0030981
Docosahexaenoic acid	-0.82248	0.0010915	1.22624	0.0034363
2,2'-Dithiobisbenzothiazole	1.0544	1.06E-05	1.315656168	9.0763e-05
Carnosic acid	-5.9712	1.32E-05	1.307462957	0.00010627
Praziquantel	0.80702	0.0043313	1.141947674	0.010595
Famotidine	4.2358	3.70E-05	1.302945984	0.00022647
Canrenone	3.3832	0.0026951	1.16241966	0.0071217
Aloxistatin	1.8436	0.0017623	1.15884582	0.0050635
Medroxyprogesterone	0.2991	0.0019945	1.202358625	0.0055891
Calycanthine	-2.9043	9.65E-05	1.276546115	0.00047317

Oxysanguinarine	1.3058	1.24E-05	1.318590813	0.00010013
Caffeoyl quinic acid; PlaSMA ID-1968	0.91035	0.0011984	1.22802966	0.0037043
Arachidoyl Ethanolamide	-0.40809	0.017079	1.058018547	0.033761
Chenodeoxycholic acid; CE0; RUDATBOHQWOJDD-BSWAIDMHSA-N	1.9458	0.017765	1.014699548	0.034913
Florfenicol	-7.0176	7.60E-13	1.353689705	3.8784e-10
tabersonine	1.104	0.011283	1.072255109	0.023926
Bergamottin	-4.4705	0.00013684	1.287851124	0.0006432
Haloxyfop	-5.86	4.91E-09	1.349252484	1.4733e-07
Coumaphos	2.7328	0.0041992	1.162240511	0.010321
Secoisolariciresinol; PlaSMA ID-2027	1.1158	0.0090807	1.086223005	0.020021
Melibiose	-2.7685	0.00015771	1.276789604	0.00071996
Coprostanone	1.4287	0.00059423	1.22751457	0.0021119
cholic acid; CE0; BHQCQFFYRZLCQQ-OELDTZBJSA-N	-2.0199	0.015345	1.038343744	0.030932
Neotame	-7.6121	2.85E-13	1.353818431	2.9118e-10
Econazole	1.1163	0.00016384	1.28429467	0.0007362
Campesterol	-6.2341	7.44E-08	1.346339253	1.4325e-06
2'-DEOXYCYTIDINE 5'-DIPHOSPHATE	3.7897	1.61E-09	1.348584029	6.8422e-08
Diacerein	-0.73135	0.019269	1.045240719	0.037436
Octaethylene glycol	0.81186	0.0028084	1.172284528	0.0073513

Ergosterol	4.4032	0.0011443	1.208394388	0.0035695
Palmitoylcarnitine	0.595	0.011268	1.071695468	0.023926
URIDINE 5'-DIPHOSPHATE	3.8427	0.00058926	1.245445743	0.0021016
Lincomycin A	-0.80088	0.0051637	1.138126495	0.012306
Miconazole	3.9266	0.00059803	1.241054748	0.002118
Glabrol	0.82589	0.015033	1.038430668	0.030546
Blasticidin S	-4.3144	0.00074663	1.225564922	0.0025217
Linoleyl Carnitine	1.4507	0.0041693	1.150995107	0.010272
veratramine	1.3338	0.00026836	1.249358168	0.0010993
Latanoprost	4.1503	0.00065018	1.229948717	0.0022628
Fipronil	-3.3528	2.40E-05	1.299536028	0.00017493
Lafutidine	-4.7396	5.18E-05	1.288971618	0.00030026
Verapamil	2.8519	0.019427	1.031665431	0.037671
glycolithocholic acid	-3.0215	0.0018423	1.206229659	0.0052236
FOLIC ACID	2.5412	2.30E-05	1.304246168	0.00016867
Apixaban	2.1312	0.021197	1.016265386	0.040337
Quercetin-3-O-glucoside; PlaSMA ID-2533	2.8386	0.002885	1.15518618	0.0074688
Obacunone	1.2942	0.0066844	1.105585841	0.015531
Flavin Mononucleotide	-6.5192	1.17E-05	1.310276859	9.7011e-05
TETRACYCLINE HYDROCHLORIDE	0.94273	0.0009689	1.224088369	0.0030981
Penoxsulam	2.264	0.00098211	1.225617985	0.0031207

Myricitrin	3.3143	0.014539	1.009997097	0.029659
glycoursodeoxycholic acid	2.4827	0.00047847	1.223750535	0.0017877
GLYCOCHOLATE	-3.0676	1.15E-05	1.308094865	9.6622e-05
Fexofenadine	-1.6928	9.49E-05	1.283851552	0.0004697
Glimepiride	0.90062	0.017695	1.037627632	0.034843
Ganoderic Acid B	1.0228	0.0048044	1.151009921	0.011585
Ticagrelor	0.85644	0.00029903	1.268521674	0.0011961
RAFFINOSE	-5.1035	0.0010352	1.230932801	0.0032792
Kutkoside	2.5649	0.0080292	1.098548293	0.018119
harringtonine	0.89569	0.015176	1.051330703	0.030713
Gossypol	-1.1549	0.00010775	1.290066813	0.00052089
Darunavir	2.4121	0.00068161	1.228076243	0.0023409
Ergosine	-3.8231	0.00016891	1.283162168	0.00075565
Cucurbitacin B	0.70433	0.023908	1.009468814	0.044826
Biliverdin	3.9237	0.0041421	1.134218757	0.01023
ETOPOSIDE	-4.4825	2.77E-08	1.343617275	5.883e-07
5-L-Glutamyl-L-cysteinylglycine	-2.8221	0.0023169	1.201678889	0.00637
Isotetrandrine	2.2809	0.015864	1.038494951	0.031727
Ioxitalamic acid	3.3022	0.00024398	1.265772298	0.0010241
Syrosingopine; PlaSMA ID-3154	1.5692	0.013985	1.047022592	0.028716
STACHYOSE	-3.935	0.00060016	1.233183317	0.0021182

THYROXINE	5.7171	0.00022653	1.267653381	0.00098324
Glyceryl trioleate	-4.0046	5.97E-05	1.302126424	0.00033472
Vincristine sulfate	2.3347	0.018083	1.027966804	0.03547
Rebaudioside C	1.2335	0.00094588	1.217988655	0.0030726

Supplementary Table 9 The differential metabolites of Model vs. PWEH.

name	log2(FC)	p.value	Vip	FDR
Urea	3.5184	6.57E-10	1.472061845	4.4678e-08
Piperidine	0.46947	0.00028055	1.310743052	0.0011006
L-ALANINE	2.1661	2.63E-06	1.418935272	2.5839e-05
Choline	-1.293	8.98E-08	1.457833906	2.1277e-06
Proline	0.58182	3.47E-05	1.368231546	0.00020137
L-Valine; CE10; KZSNJWFQEVHDMF-BYPYZUCNSA-N	0.2842	0.018785	1.021870141	0.042394
PHENYLETHANOLAMINE	0.7131	1.55E-05	1.395654407	0.00011005
Taurine	1.7867	1.82E-07	1.448513767	3.4427e-06
L-Pyroglutamic acid; CE0; ODHCTXKNWHHXJC-VKHMYHEASA-N	-1.1614	0.00011029	1.338614985	0.00053384
N-Methyl-L-proline; CE0; CWLQUGTUXBXTLF-YFKPBYRVSA-N	1.3525	1.24E-05	1.388622329	9.1262e-05
Pipecolic acid	0.812	0.00020847	1.32622933	0.00086386
Creatine	2.4033	2.16E-09	1.469792518	1.2212e-07
Isoleucine	0.3384	0.00023488	1.319331358	0.00094305
Thiazolidine-4-carboxylic acid	5.0948	9.39E-06	1.411273415	7.31e-05

CREATININE	2.493	3.07E-08	1.457923672	1.0444e-06
2-Aminobenzoic acid; CE0; RWZYAGGXGHYGMB-UHFFFAOYSA-N	4.245	3.22E-05	1.379745152	0.00019109
N-METHYLGLUTAMATE	-5.3818	1.35E-07	1.45471605	2.8652e-06
1-Naphthylamine	-1.6722	0.00046823	1.304982244	0.0016469
Proline betaine; CE0; CMUNUTVVOOHQPW-LURJTMIESA-N	2.4644	5.35E-08	1.458829806	1.4654e-06
Indole-3-carboxyaldehyde	-1.5984	0.0004387	1.305507247	0.0015756
SPERMIDINE	1.3037	2.40E-05	1.38329621	0.00015186
LYSINE	0.7155	5.29E-05	1.368694631	0.00029169
L-Glutamic acid; CE0; WHUUTDBJXJRKMK-VKHMYHEASA-N	0.73003	0.00017029	1.327022295	0.00075194
D-Xylulose	6.0544	6.19E-06	1.415202691	5.1316e-05
Vanillin	3.2483	1.82E-07	1.445682873	3.4427e-06
4-Aminosalicylic acid; CE0; WUBBRNOQWQTFEX-UHFFFAOYSA-N	3.461	0.00084847	1.252470021	0.0027474
UREIDOPROPIONATE	5.8004	7.64E-06	1.411734277	6.1398e-05
Allantoin; CE0; POJWUDADGALRAB-UHFFFAOYSA-N	1.7504	3.79E-08	1.456471215	1.1711e-06
1-Benzylimidazole	-1.3653	9.90E-05	1.357494094	0.00049028
PHENYLALANINE	0.41183	1.75E-05	1.391195614	0.00011949
Uric acid; CE10; LEHOTFFKMJEONL-UHFFFAOYSA-N	-3.5316	3.26E-06	1.419268012	3.0548e-05
3-METHYL-2-OXINDOLE	-2.057	0.0096276	1.119054882	0.023893

ADENINE	-2.3174	1.31E-05	1.383276856	9.5147e-05
1-Methyl-4-phenyl-1,2,3,6-tetrahydropyridine	3.7843	9.18E-08	1.453390146	2.1277e-06
ARABITOL	1.8387	0.00067997	1.263991127	0.002274
2,4-Toluene diisocyanate	1.2242	0.0013071	1.228519568	0.0041211
P-OCTOPAMINE	-1.6834	6.43E-05	1.351101595	0.0003451
CITRULLINE	3.3027	1.97E-07	1.451645072	3.5878e-06
Herniarin	2.7704	0.00037611	1.289530664	0.00139
HISTIDINE	0.90243	2.21E-05	1.384930832	0.00014272
Diallyl Trisulfide	-3.8341	0.00013095	1.343359361	0.00060165
Tyrosine	-0.95578	1.89E-05	1.391272235	0.00012789
4-PYRIDOXATE	6.087	5.36E-07	1.438028565	7.2941e-06
Methyprylon	-0.68041	0.0027571	1.192572844	0.0079443
Selegiline	-2.1049	1.92E-05	1.394204767	0.00012914
THIOPURINE S-METHYLETHER	-3.811	0.00070232	1.287340247	0.0023183
Kynurenic acid	8.5895	2.07E-08	1.461067412	8.0706e-07
4-Nitroquinoline-1-oxide	-1.1576	0.0002224	1.332794952	0.00091105
5-Hydroxy-3-indoleacetic acid	3.2523	0.0015565	1.232304967	0.0047831
Phenylacetylglycine	4.5359	3.98E-05	1.365405592	0.00022537
3,4-DIHYDROXY-L-PHENYLALANINE	3.8801	7.66E-05	1.349634598	0.00039437
N-FORMYL-L-METHIONINE	0.64536	0.00068815	1.265962885	0.0022938

Cysteine-S-sulfate; CE0; NOKPBJYHPHHWAN-REOHCLBHSA-N	2.1788	2.15E-05	1.389368655	0.00013947
HIPPURATE	3.7387	0.00017846	1.329726142	0.00078126
RICININE	1.0443	0.00038428	1.295250253	0.0014049
GALACTOSE	-3.0979	1.63E-06	1.423669986	1.7153e-05
Tryptophan	-2.2123	0.0021815	1.224201611	0.0064874
Phenylethylmalonamide	3.8777	0.00033435	1.315719388	0.0012678
N-ACETYL-L-PHENYLALANINE	4.1866	0.00011339	1.340383516	0.00054558
9,10-Phenanthrenedione	3.3566	0.00017496	1.330324305	0.00076922
1,1,2-Trimethyl-1H-benzo[e]indole	-4.4777	1.07E-05	1.393518801	8.0479e-05
N-Acetylglutamine	4.1276	9.52E-10	1.472137571	5.7112e-08
Juglone	-4.4507	3.74E-07	1.447345822	5.695e-06
D-Pantothenic Acid	3.3661	1.64E-07	1.451682296	3.2874e-06
4-O-Methylphloracetophenone	-1.71	9.96E-07	1.442231956	1.1681e-05
alpha-oxo-1h-indole-3-propanoic acid	-6.2776	4.76E-07	1.441813801	6.566e-06
Citric acid	-3.2506	1.42E-07	1.447471568	2.9656e-06
BUTYL PARABEN	3.3492	0.00020161	1.315334762	0.00084471
Methazolamide	-1.064	0.0020277	1.226224222	0.0060476
Felbamate	3.1922	0.00019869	1.315772056	0.00084471
L-Cystine	4.0806	4.10E-05	1.379019751	0.00023081
L-Anserine; CE0; MYYIAHXIVFADCU-QMMMGPOBSA-N	1.1942	8.61E-05	1.347422662	0.00043334

Uridine; CE0; DRTQHJPVMGBUCF-XVFCMESISA-N	2.4398	6.31E-06	1.405473343	5.1895e-05
Patchouli alcohol	-2.1162	4.48E-07	1.446025903	6.4327e-06
Pinacidil	1.0472	3.34E-05	1.386634129	0.00019603
Isoleucylaspartate	5.4749	1.02E-05	1.402473365	7.8129e-05
Genipin	-2.6359	0.00012365	1.356415023	0.00057589
Kainic Acid	3.2751	0.00080073	1.265251823	0.0026011
Daidzein; PlaSMA ID-1090	2.2325	0.0099132	1.092360158	0.024365
Chrysin	1.624	0.0099998	1.076269449	0.024517
sn-Glycero-3-phosphocholine	-2.1568	1.68E-06	1.43104645	1.7456e-05
Diaveridine	-0.46076	0.00051398	1.278330399	0.0017893
falcarindiol	0.63274	0.0018441	1.207743983	0.0056149
Vitamin B1	1.9268	6.48E-05	1.368267126	0.00034591
Magnolol; PlaSMA ID-1190	6.5262	3.12E-06	1.4195103	3.0036e-05
Azacyclonol	1.335	2.72E-05	1.376518476	0.00016786
ellipticine	5.8026	4.30E-06	1.423413916	3.8177e-05
Linolenic acid	-3.492	1.10E-06	1.430844567	1.252e-05
Laurocapram	0.79966	0.0045523	1.160332901	0.012382
XANTHOSINE	-5.9373	8.39E-07	1.439776589	1.0309e-05
Diazoxon	-5.0392	4.35E-13	1.479123193	6.3416e-11
Thiamethoxam	4.4441	3.34E-05	1.382913011	0.00019603

6-PHOSPHOGLUCONATE	7.501	3.48E-08	1.462960918	1.1312e-06
D-Sphingosine	0.84382	0.001979	1.218509914	0.0059545
Retinoic acid	2.6306	0.022639	1.002689129	0.049723
(9Z)-9-octadecenoic acid	-1.018	0.020824	1.014992924	0.04658
Arachidonic Acid	-1.0692	0.022825	1.000908086	0.049854
Fructoselysine	1.4492	0.00011043	1.351010344	0.00053384
gamma-Glutamyltyrosine	-1.4457	0.011585	1.056133885	0.027739
Loureirin B	0.33196	0.020934	1.026570029	0.046723
steviol	4.4316	0.00070914	1.276740255	0.0023333
Valaciclovir	1.8483	0.00038406	1.303533541	0.0014049
Sulcotrione	-3.862	3.55E-12	1.478163263	4.5266e-10
xylan	-5.9698	1.63E-06	1.433376454	1.7153e-05
Aflatoxin G1	2.9235	0.0005654	1.276995321	0.0019418
Tulipinolide	0.91044	0.0019453	1.201053023	0.0058705
Docosahexaenoic acid	0.52493	0.015353	1.042057925	0.035349
2,2'-Dithiobisbenzothiazole	3.8776	5.56E-08	1.457947366	1.4654e-06
Carnosic acid	6.1454	8.71E-07	1.435374212	1.0575e-05
Famotidine	-4.1756	1.44E-06	1.439383191	1.5492e-05
D-ribo-Phytosphingosine	0.58871	0.0089436	1.12121881	0.022324
Canrenone	-2.7405	0.00088947	1.263915387	0.0028711
Oxysanguinarine	-1.5218	8.67E-09	1.465446951	4.4241e-07

Arachidoyl Ethanolamide	0.76454	0.00020937	1.328157268	0.00086386
Florfenicol	5.8171	2.10E-06	1.423851056	2.1188e-05
Cilastatin	0.63439	0.0032982	1.181807737	0.0092932
Bergamottin	2.2246	0.0088722	1.10320304	0.022235
Haloxyfop	4.7368	1.01E-05	1.406047918	7.7693e-05
Coumaphos	-2.4363	0.0010886	1.261310239	0.0034918
Coprostanone	-1.5558	0.0048828	1.15539874	0.01314
Epicholestanol	-2.1104	0.0064715	1.105760003	0.016969
cholic acid; CE0; BHQCQFFYRZLCQQ-OELDTZBJSA-N	2.1335	0.0066107	1.139239558	0.017245
Neotame	5.2982	1.72E-06	1.422946112	1.7738e-05
Melibiose	3.4539	0.00073014	1.272132326	0.0023871
Campesterol	1.5448	0.0215044	1.005101074	0.047682
2'-DEOXYCYTIDINE 5'-DIPHOSPHATE	-4.4431	4.50E-11	1.476812971	4.1745e-09
Diacerein	0.93438	0.00029722	1.300052855	0.0011527
Ergosterol	-3.6186	0.00035013	1.309985606	0.001313
Palmitoylcarnitine	-0.79597	0.00070125	1.283837516	0.0023183
7-alpha-hydroxy-4-cholesten-3-one	-0.73299	0.0013009	1.258105473	0.0041211
URIDINE 5'-DIPHOSPHATE	-3.2752	0.0033075	1.189091655	0.0092937
Miconazole	-4.3074	2.49E-05	1.394887502	0.00015586
Glabrol	-0.66674	0.0109998	1.09061633	0.026645

Blasticidin S	2.8072	0.00199	1.213493692	0.00597
Linoleyl Carnitine	-1.5939	0.00036926	1.315226662	0.0013746
Oleoyl-L-Carnitine	-0.78707	0.014349	1.069292768	0.033415
Latanoprost	-3.5609	0.00012917	1.344553031	0.00059618
Fipronil	4.2002	8.20E-07	1.440258387	1.0309e-05
Phlorhizin	0.30086	0.015216	1.05514245	0.035114
Lafutidine	0.78217	0.0033452	1.197661014	0.0093738
Verapamil	-2.3365	0.0091336	1.122771008	0.022723
glycolithocholic acid	1.9984	9.20E-05	1.360211771	0.0004602
FOLIC ACID	-2.4235	1.02E-06	1.435485677	1.1852e-05
Quercetin-3-O-glucoside; PlaSMA ID-2533	-2.6959	0.00045949	1.286615948	0.0016241
Flavin Mononucleotide	7.0881	1.24E-07	1.455746754	2.7395e-06
Penoxsulam	-2.5062	3.06E-05	1.388314194	0.00018604
Myricitrin	-6.612	4.23E-05	1.379651297	0.00023681
glycoursodeoxycholic acid	-1.6687	0.00031736	1.284821562	0.0012169
GLYCOCHOLATE	-2.0625	0.0027133	1.2117622	0.0078393
Fexofenadine	1.8238	4.46E-06	1.411509721	3.9217e-05
RAFFINOSE	7.9303	4.19E-07	1.444537747	6.1132e-06
Gossypol	1.861	1.96E-05	1.387133011	0.00013031
cynarin	-2.5113	0.0048953	1.129813854	0.01314
Ergosine	3.0671	1.97E-05	1.381641654	0.00013031

Biliverdin	-3.1939	0.0051119	1.147062121	0.013686
ETOPOSIDE	2.8326	0.00069591	1.270863526	0.0023121
5-L-Glutamyl-L-cysteinylglycine	1.6403	0.0026007	1.190159832	0.007576
Ioxitalamic acid	-3.7428	0.0002032	1.346442072	0.00084599
Isotetrandrine	-1.2589	0.0061518	1.130771281	0.016172
Syrosingopine	-1.9344	1.09E-06	1.440932503	1.2475e-05
Saquinavir	-1.5948	0.0132995	1.069344126	0.031175
STACHYOSE	4.2191	1.84E-05	1.395375878	0.00012479
THYROXINE	-5.2625	2.27E-05	1.402896884	0.00014552
Rebaudioside C	-3.0863	0.00015936	1.339446689	0.00070981
Madecassoside; PlaSMA ID-3491	-2.9404	0.0014378	1.253713373	0.0044986
Tomatine	-1.2727	0.0100023	1.0968036	0.024517
